# Optical Interrogation of Sympathetic Neuronal Effects on Macroscopic Cardiomyocyte Network Dynamics

**DOI:** 10.1016/j.isci.2020.101334

**Published:** 2020-07-01

**Authors:** Rebecca-Ann B. Burton, Jakub Tomek, Christina M. Ambrosi, Hege E. Larsen, Amy R. Sharkey, Rebecca A. Capel, Alexander D. Corbett, Samuel Bilton, Aleksandra Klimas, Guy Stephens, Maegan Cremer, Samuel J. Bose, Dan Li, Giuseppe Gallone, Neil Herring, Edward O. Mann, Abhinav Kumar, Holger Kramer, Emilia Entcheva, David J. Paterson, Gil Bub

**Affiliations:** 1 University of Oxford, Department of Pharmacology, Mansfield Road, Oxford OX1 3QT, UK; 2 University of Oxford, Department of Physiology, Anatomy and Genetics, British Heart Foundation Centre of Research Excellence, Parks Road, Oxford OX1 3PT, UK; 3 The George Washington University, Department of Biomedical Engineering, Washington, DC 20052, USA; 4 University of Exeter, Physics and Astronomy, Exeter EX4 4QL, UK; 5 Department of Computational Molecular Biology, Max Planck Institute for Molecular Genetics, Ihnestraße 63-73, 14195 Berlin, Germany; 6 University of Oxford, Department of Biochemistry, Glycobiology Institute, Oxford, UK; 7 McGill University, Department of Physiology, McIntyre Medical Sciences Building, Room 1128, 3655 Promenade Sir William Osler, Montréal, QC H3G 1Y6, Canada

**Keywords:** Optical Imaging, Neuroscience, Techniques in Neuroscience

## Abstract

Cardiac stimulation via sympathetic neurons can potentially trigger arrhythmias. We present approaches to study neuron-cardiomyocyte interactions involving optogenetic selective probing and all-optical electrophysiology to measure activity in an automated fashion. Here we demonstrate the utility of optical interrogation of sympathetic neurons and their effects on macroscopic cardiomyocyte network dynamics to address research targets such as the effects of adrenergic stimulation via the release of neurotransmitters, the effect of neuronal numbers on cardiac behavior, and the applicability of optogenetics in mechanistic *in vitro* studies. As arrhythmias are emergent behaviors that involve the coordinated activity of millions of cells, we image at macroscopic scales to capture complex dynamics. We show that neurons can both decrease and increase wave stability and re-entrant activity in culture depending on their induced activity—a finding that may help us understand the often conflicting results seen in experimental and clinical studies.

## Highlights

• A methodology to study neuron-cardiac interactions at multicellular/tissue level
• Cardiac sympathetic stellate neurons can alter cardiac wave stability
• Optogenetic control of sympathetic neurons offers temporal and spatial control
• Increasing neuron density in co-cultures affects cardiac firing frequency

## Graphical Abstract

**Figure:**
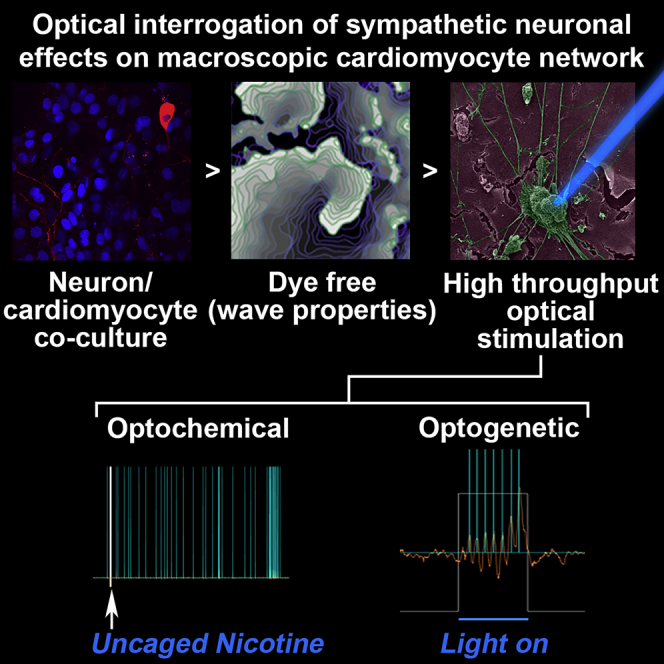

## Introduction

Cardiac impulse formation and conduction are modulated by autonomic activity, and the autonomic nervous system plays an important role in the initiation and maintenance of arrhythmias in diseased hearts (Bub and Burton, 2014; Herring et al., 2019; Winfree, 1987). Sympathetic nerves release noradrenaline, which activates cardiac β-adrenergic receptors to modulate myocyte repolarization and calcium handling via alterations of transmembrane currents and intracellular calcium homeostasis (Bers, 2008; Zaccolo and Pozzan, 2002). Increased sympathetic activity, which can occur during epileptic seizures (Devinsky, 2004) and is also associated with chronic diseases such as hypertension (Julius, 1998) and heart failure (Cohn et al., 1984), is often associated with increased risk of re-entrant arrhythmias (Chen et al., 2014). Tissue damage can also alter the distribution of innervation where cardiac cell death following myocardial infarction causes sympathetic denervation followed by nerve sprouting and reinnervation (Gardner et al., 2016).

Nerve sprouting may promote the heterogeneity of excitability and refractoriness, which was suggested as a mechanism for increased arrhythmia susceptibility in the reinnervated infarct border zone (Cao et al., 2000; Chen et al., 2001). However, recent clinical studies (Boogers et al., 2010; Fallavollita et al., 2014, 2017; Nishisato et al., 2010; Standen et al., 1989; Vaseghi et al., 2012) have shown that cardiac sympathetic denervation (rather than reinnervation) can lead to a higher risk of ventricular arrhythmias and arrhythmic death. Experimental and computational studies linked the beneficial effect of innervation to attenuation of infarct-induced vulnerability to repolarization alternans via β-adrenergic activation (Tomek et al., 2017, 2019) or to reduction of electrophysiological heterogeneity and calcium mishandling, which was present even when the nerves were not activated (Gardner et al., 2015). Resolving the unclear pro- or antiarrhythmic effect of post-infarction reinnervation may also involve the precise understanding of neural heterogeneity and its role in arrhythmia modulation. Research on these questions may therefore benefit from the use of a cell culture model system where the effects of innervation can be precisely controlled. Co-cultures of cardiac myocytes and sympathetic neurons have been investigated for over 30 years (Furshpan et al., 1976; Horackova et al., 1993); however, these studies were carried out at microscopic (single cell) scales where arrhythmogenicity cannot be directly assessed. Tissue heterogeneity and impulse conduction velocity (CV) play key roles in the initiation and stability of re-entrant spiral waves (Winfree, 1987). Although CV depends in part on the excitability of individual myocytes, it also depends on cell-cell connectivity and tissue heterogeneity (Kleber and Rudy, 2004; Shaw and Rudy, 1997).

Confluent myocyte monocultures imaged at macroscopic space scales have allowed the investigation of more complex functional tissue level properties such as wave propagation and pattern formation (Entcheva and Bien, 2006; Tung and Zhang, 2006), and their ability to support reentrant spiral waves has validated their use as a model of arrhythmogenesis. Optical mapping of these cultures has given an insight into important arrhythmogenic mechanisms, including unidirectional conduction block, junctional coupling, and remodeling (Tung and Zhang, 2006). Confluent co-cultures of myocytes and neurons imaged at macroscopic scales (>1 cm^2^) are a potentially useful biological model system for the study of the proarrhythmic effects of abnormal sympathetic activation on cardiac conduction.

Recent studies have used optogenetic approaches to spatially control sympathetic activation to gain insights on communication dynamics between cardiomyocytes and neurons (Prando et al., 2018). In addition, a number of studies have quantified sympathetic axon density in healthy and diseased myocardium (Clarke et al., 2010; Ieda et al., 2007; Muhlfeld et al., 2010; Zhou et al., 2004), highlighting the relevance of neuron numbers and sympathetic miswiring in the diseased heart (Freeman et al., 2014). In this work, we report the first macroscopic optical mapping measurements of cardiac monolayers co-cultured with cardiac sympathetic stellate neurons imaged using our recently published dye-free optical imaging method (Burton et al., 2015). Further, in addition to the dye-free imaging experiments, we use another approach involving optogenetics combined with an automated system for high-throughput all-optical imaging as demonstrated in (Klimas et al., 2016, 2020) (OptoDyCE) to relate physical neuron-myocyte contacts to functional coupling between these cell populations and also quantify how neural stimulation modulates cardiac behavior, which may ultimately give insights pertinent to pathophysiological questions. Finally, we explored the effects of neuron numbers on cardiac behavior and their ability to modulate cardiac excitability.

## Results

### 1] Stellate Sympathetic Neurons Make Contacts with Cardiomyocytes

**Figure 1:**
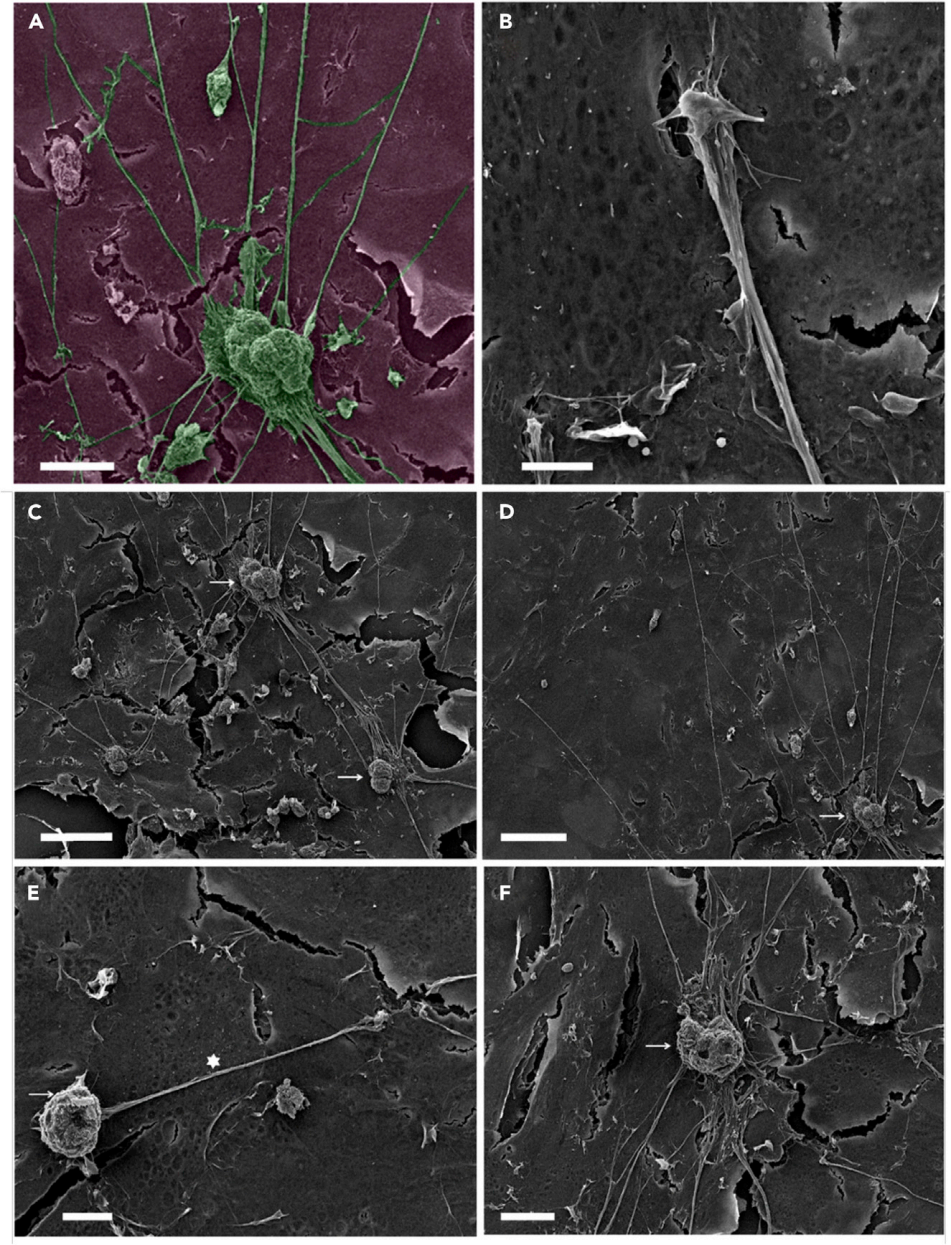
Scanning Electron Microscopy of Cardiac-Neuron Co-cultures (A) Exemplar image for sympathetic neurons growing in co-culture with cardiomyocytes *in vitro* (micrographs were colored in post-processing). Scale bar: 25 μm. (B) Close up of a connection between neurite extension and cardiac syncytium showing connections between neurons and myocytes. Scale bar: 5 μm. (C–F) SEM images showing stellate sympathetic neurons on cardiac monolayers. Neurons making connections to other neurons (C), wide spreading neurite extensions (D), neuron bodies and extensions make contact with myocytes (E), white asterisk showing dendritic process (E) and white arrow (F) indicating neuron body. Scale bars: (C) 50, (D) 50, (E) 10, and (F) 20 μm respectively. See also Figure S1.

Scanning electron microscopy (SEM) of sympathetic neurons growing in co-culture with cardiomyocytes *in vitro* shows connections between neurite extension and cardiac syncytium (Figure 1A). The neuron bodies and extensions clearly make physical contact with myocytes. Close up of a connection between neurite extension and cardiac syncytium shows connections between neurons and myocytes (Figure 1B). Figures 1C–1F demonstrate that neuron bodies and extensions make contact with myocytes (white asterisk showing dendritic process, Figure 1E; white arrow indicating neuron body, Figure 1F). See also Figure S1 for wide-field SEM images showing extensive dendritic processes and arborization. Immunofluorescence staining of co-cultures demonstrated that sympathetic neurons showed positive staining for tyrosine hydroxylase (TH) (Figures S2 and S4), and fibroblast contamination in the co-cultures was assessed by staining with vimentin (Figure S3), which showed low abundance.

### 2] Neurite Extensions in Co-cultures of Sympathetic Neurons and Cardiomyocytes

**Figure 2:**
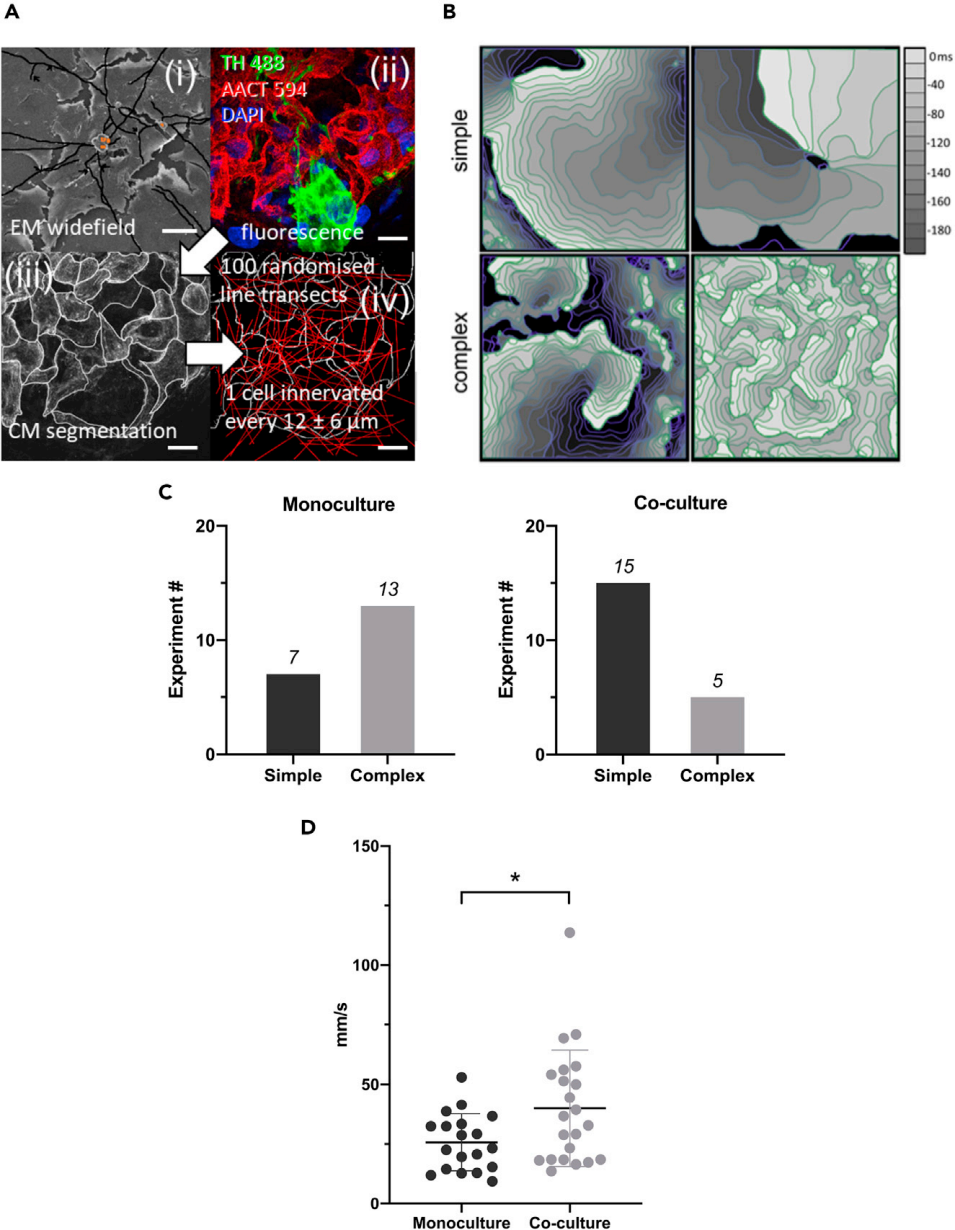
Structure-Function Relationship Between Cardiac Myocytes and Neurons (A) (i) Wide-field scanning electron microscopy image of neuron cell bodies (square orange overlay) showing manual segmentation of dendritic processes traversing the CM monolayer (black lines). See Figure S1 for further images. (ii) Single slice from multichannel confocal stack of CM cells in monolayer (red) around a cell body (green) with nuclei (blue). (iii) Maximum intensity projection of CM channel from (ii) was manually segmented to highlight cell boundaries (white lines). (iv) Randomized linear trajectories were taken through the segmented image to identify the average distance between cell boundaries, indicating the total number of cells that can be innervated by a given length of dendritic process. Scale bars: (i) 50 μm; (ii)–(iv) 20 μm. (B) Wave dynamics measured by dye-free imaging in Oxford monocultures and co-cultures (see also Figure S5). Isochronal maps of wave dynamics in confluent cardiac-stellate neuron co-cultures display a variety of complex rhythms similar to those seen in intact hearts. Wave dynamics here are classified as simple (top left: targets or top right: single spirals) or complex (bottom left: multiple spiral waves or bottom right: wavelets of activity). (C) Monocultures display more complex dynamics than co-cultures, which display predominantly simple wavefronts with few wave breaks (p < 0.05, Chi-square). (D) Comparison of 90-percentile of wave speed for monocultures (25.73 ± 11.88 mm/s, n = 19) and co-cultures 39.96 ± 24.37 (n = 22). Normal distribution of the data was confirmed using the Kolmogorov-Smirnov test, and data were compared using unpaired, two-tailed t-test (^∗^ indicates p = 0.026). Horizontal bars in D indicate data means ± stdev. See Figure S13 for wave speeds in SBU cultures.

Release from varicosities along the length of the dendrites would enable synchronized signaling to the myocytes. Neurite lengths were segmented, tabulated, and measured (Figures 2A(i) and S1). The average neurite length is computed as a weighted average: 1183.07 ± 375.42 μm. To estimate the average number of cardiac cell boundaries a dendritic process might encounter, we performed image analysis in a confocal image stack of fluorescently labeled cardiomyocytes (TH488) (Figure 2A(ii)). A maximum intensity projection was taken of the image stack before manually segmenting the cardiomyocyte (CM) cell boundaries (Figure 2A(iii)). One hundred random transects were calculated for the segmented image shown in Figures 2A(iii) and 2A(iv). The average number of cell boundaries encountered along these 50 μm trajectories were N = 4.21 ± 2.05. This provides an estimate of the distance between cell boundaries as being 50 μm/N = 11.87 ± 5.77 μm. The simulation shows that a cell boundary is crossed every 12 ± 6 μm. For ∼1,000 μm of total neuron process length this corresponds to 1,000/(12 ± 6) = 56–167 cell boundaries crossed i.e. ∼ a maximum of 56–167 myocytes innervated.

### 3] Wave Pattern Formation is Affected by the Presence of Neurons

Using off-axis dye-free imaging, we investigated how neuronal activation modulates cardiac patterns of activation in monolayer culture (Figure S5). Functional measurements were performed on the sample from day 5 onwards. We chose experimental conditions that spontaneously yield a wide variety of wavefront topologies within the imaging system's 16 × 16 mm field of view.

Introduction of an additional cell type can potentially introduce heterogeneities that would impact wave front stability. Surprisingly, co-cultures displayed fewer wave breaks than their monoculture counterparts at similar plating densities (Figures 2B and 2C), i.e. neurons had stabilizing effect on cardiac wave dynamics. We broadly classified wave dynamics as simple [periodic target waves Figure 2B (top left) and single spiral wave reentry 2 B (top right)], or complex [single dominant spiral with additional irregular waves 2 B (bottom left) and multiple equally sized wavelets 2 B (bottom right)]. Although monocultures frequently displayed complex dynamics, co-cultures rarely displayed this behavior (Figure 2C, p < 0.05, chi-square test). Indeed, we observed wavelet reentry in only one of the co-culture preparations (6 isolations, 20 preparations, where the number of samples n = 20 tested here refers to individual petri dishes; these were obtained from at least 6 separate cell isolations).

### 4] Conduction Velocity in Spontaneously Active Oxford Co-cultures is Faster Than Myocyte Monocultures

We measured conduction velocity in unstimulated co-cultures (n = 22) and myocyte monocultures (n = 19). Figure 2D shows data representing the 90-percentile of wave speed from each recording. The conduction velocity in the co-cultures was significantly faster than in myocyte monocultures. Overall, the mean conduction velocity (± stdev) in the myocyte monocultures was 25.73 ± 11.88 mm/s and 39.96 ± 24.37 mm/s in the co-cultures. To try and understand the molecular mechanisms, we conducted quantitative label-free proteomics analysis on the myocyte and co-cultures (Tables S1 and S2, Figures S8 and S10). We found changes in pathways regulating gap junction protein expression along numerous changes in pathways associated with metabolism and development (Figures S8 and S10). Additional measurements of Connexin43 (Cx43) levels in cultures using western blot technique (Figure S12) confirmed that Cx43 was higher in the co-cultures (two independent experiments).

Interestingly, in better-connected Stony Brook University (SBU) co-cultures that were not spontaneously active/arrhythmic, the stimulated conduction velocities were very similar at 1 Hz electrical pacing for different neuron concentrations (Figure S13), showing no significant difference using ANOVA followed by Tukey-Kramer.

### 5a] Nicotine Stimulation Increases Beat Rate in Co-cultures Displaying Target Patterns

**Figure 3:**
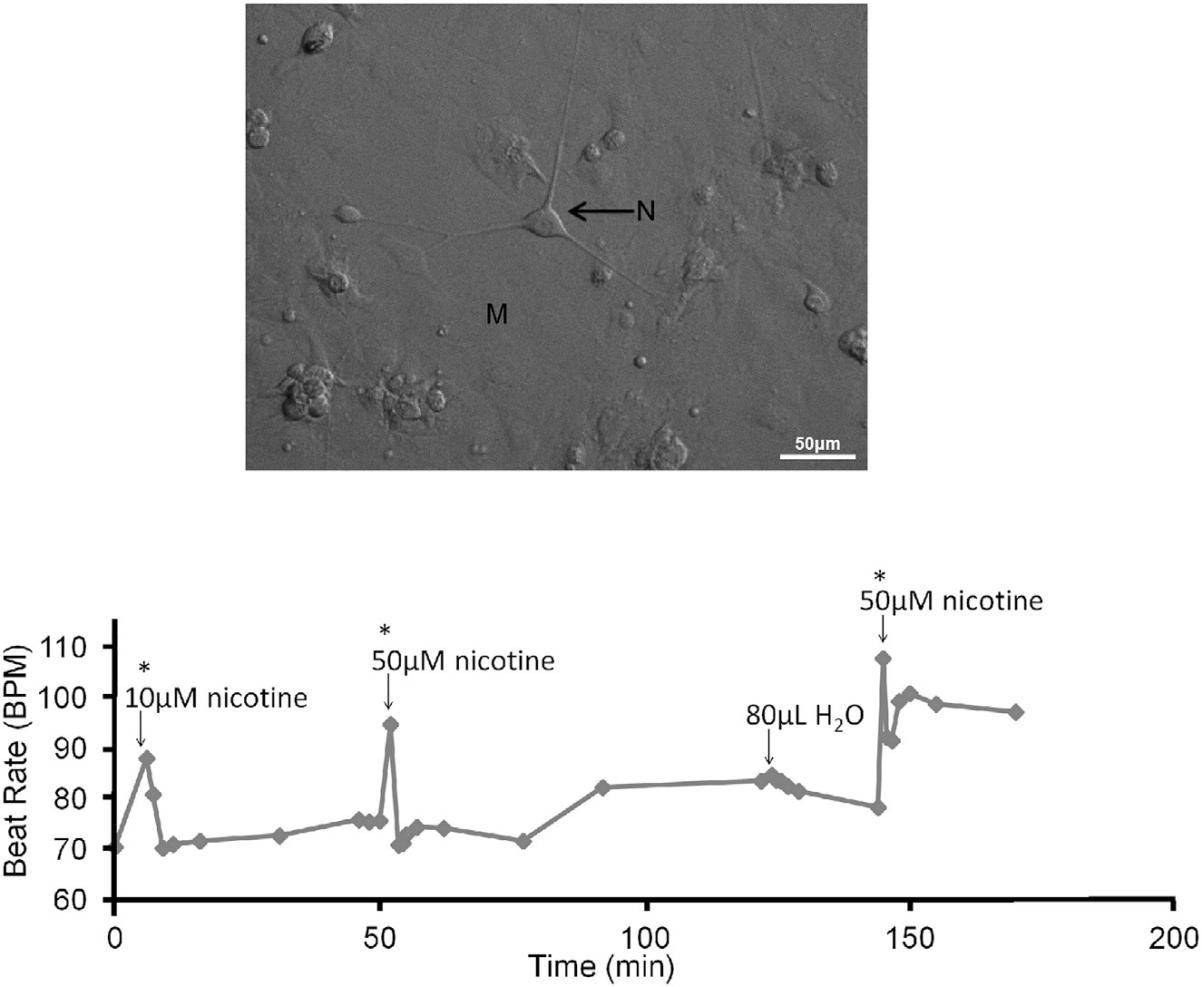
Cardiac Monolayer Response to Stellate Sympathetic Neuron Stimulation via Nicotine (Top panel) Bright field image of a confluent cardiac monolayer with cardiac sympathetic stellate neurons seeded on top. (Bottom panel) An example trace of a co-culture nicotine stimulation experiment. Each data point represents a 5-s video recording consisting of at least six full contractions. Repeat nicotine doses (10 and 50 μM) caused transient increases in myocyte beat rate; control vehicle (distilled water) of the same volume had no effect. Comparisons between pre- and post-nicotine beat rates were performed using a t test; ∗p < 0.0001.

To confirm the formation of functional connections between cardiac myocytes and sympathetic stellate neurons (Figure 3 top panel), we stimulated the cardiac myocyte contraction rate through the activation of neurons with nicotine (n = 6), in a similar fashion to the way this was done in (Shcherbakova et al., 2007). In the co-cultures we observed an average increase in beat rate of 31% ± 4 (standard error) when 10 μM of nicotine was added (up to 5 min post-nicotine addition and then the beat rate returned to baseline, Figure 3 bottom panel). We also performed (i) control experiments in which nicotine was added to cardiac monocultures (with no neurons present) and we did not see any changes in the contraction rate (p > 0.05); (ii) vehicle experiments in co-cultures (i.e. using distilled water equivalent to the quantity of nicotine used) show no increase in beat rate.

### 5b] Bath Application of Nicotine Produces a Marked Increase in Spontaneous Firing of Action Potentials in Co-cultured Neurons

**Figure 4:**
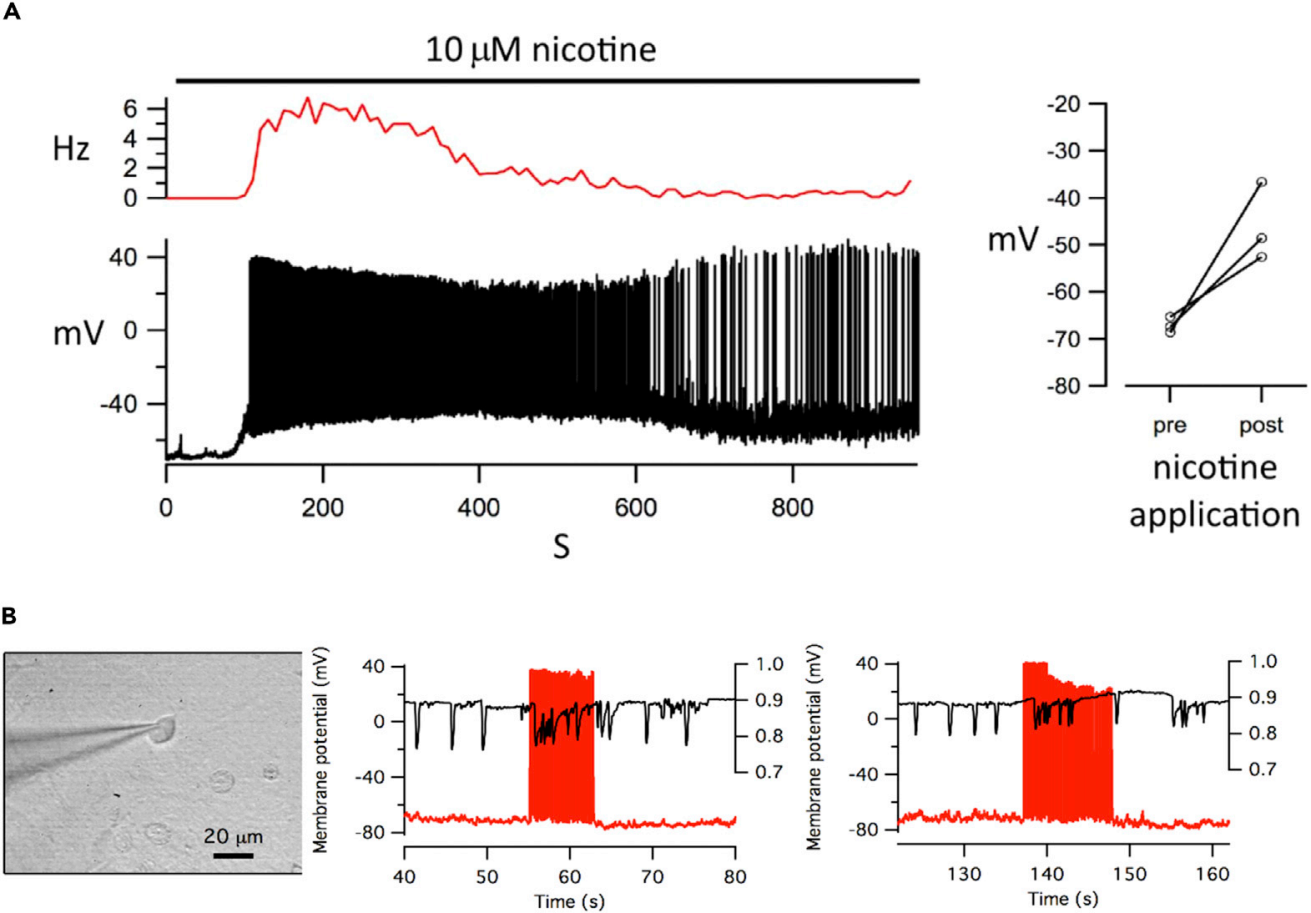
Simultaneous Electrophysiological Measurement of Neuron Membrane Potential and Video Imaging of Cardiac Dynamics (A) The bath application of nicotine to co-cultures produced membrane depolarization and a marked increase in the spontaneous firing of action potential in a patched neuron. (p < 0.05, paired t test, n = 3). The side panel shows the net depolarization of the resting potential of neurons before and after nicotine application. (B) Electrophysiological stimulation of one neuron can drive induced arrhythmia in co-cultures. Graphs show two examples in which injection of current into a patch-clamped neuron (red trace) changes cardiac behavior from normal beating to arrhythmia (black trace). Myocyte beat rate is shown by the downward deflections of the black trace, using frame cross-correlation of each video frame correlated to the original image in order to map changes. Synchronization of video imaging and electrophysiological trace is accurate to +/−2 s. See also Figure S6.

Using the whole-cell current clamp method we observed repeated membrane depolarization events (in three separate experiments) on the addition of 10 μM nicotine (Figure 4A). We measured the membrane potential of individual neurons within the co-culture at 0–60 s (pre) to 300–360 s (post) nicotine application and found a significant rise in membrane potential (p < 0.05, paired t -test). Figure S6 shows individual frames from a video recorded before and after nicotine application. The video was processed to show motion transients (white) as described in the supplement. Changes in cardiac macroscopic activity correlate with neuronal bursting following nicotine application.

### 6] One Neuron Potentially Stimulates a Connected Monolayer of One Hundred Thousand Myocytes

Pilot electrophysiology experiments (n = 3) suggested that a single neuron could potentially modulate the activity of many connected cardiomyocytes (Figure 4B). Current injection into a patched neuron caused a rapid activation of the neuron, which was correlated to irregular activity in the monolayer by calculating the cross-correlation between sequential frames in order to map changes. Electrophysiological measurement of neuron activity in co-cultures poses challenges such as trying to keep a patch on a neuron after stimulating a “beating” cardiac monolayer. These observations formed the motivation for extensive optogenetic and optochemical-based experiments that follow.

### 7] Optogenetic and Optochemical Neuronal Stimulation and the Effect of Neurotransmitter Release on Cardiomyocytes Using High-Throughput Fluorescence Methods

**Figure 5:**
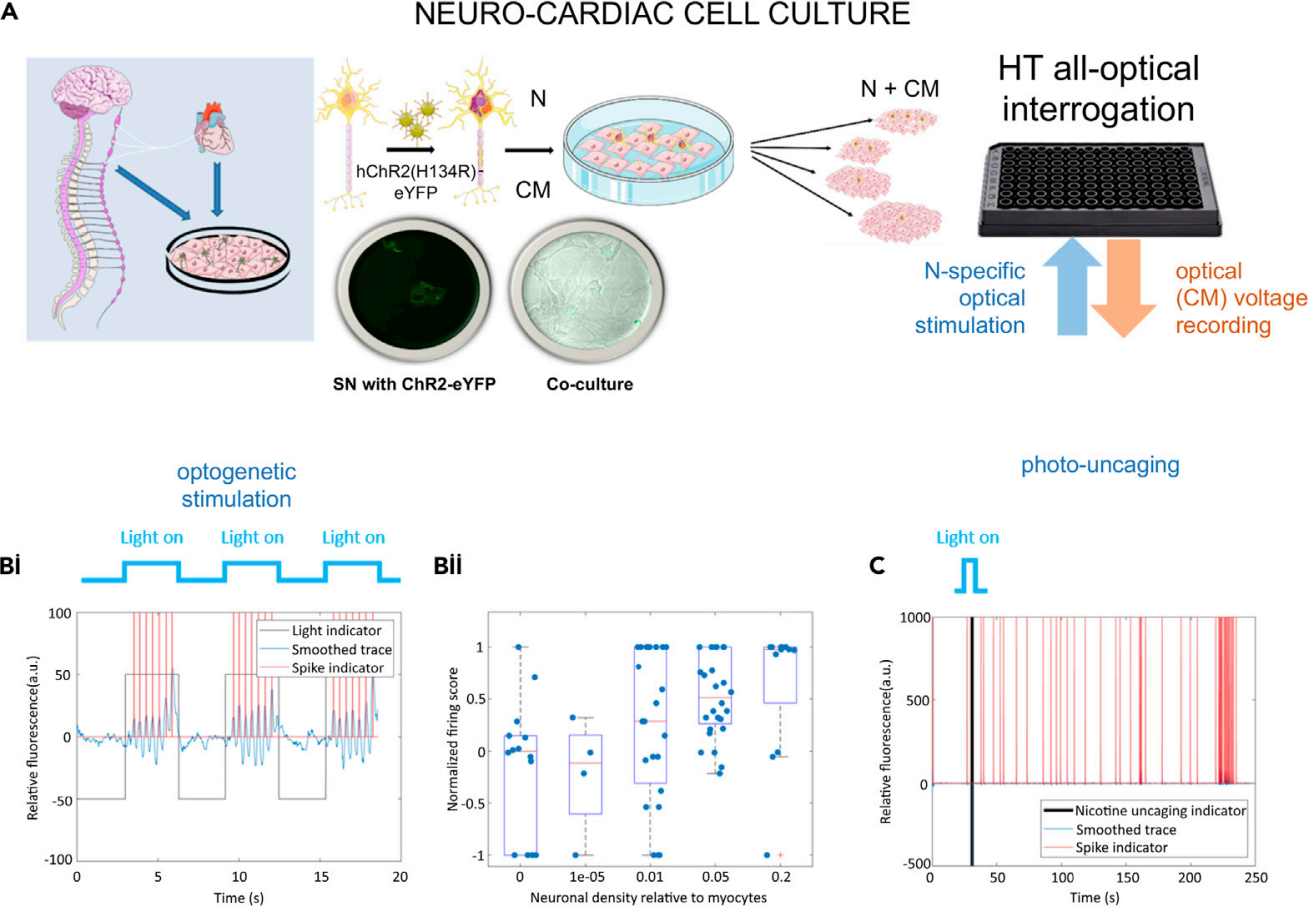
High Throughput All Optical Interrogation of Neurocardiac Cultures (A) Schematic representation of the *in vitro* co-culture system, neonatal rat cardiac stellate sympathetic neurons, and neonatal rat ventricular cardiac confluent monolayers cultured to test sympathetic-cardiac interactions. The stellate sympathetic neurons were infected with hChR2-eYFP. Schematic representation of the co-cultures with different neuron dosing regimes (neuron-myocyte ratios in 1:5, 1:20, 1:100, 1:100,000). (B and C) “Optoelectric” versus “optochemical” stimulation of neurons. (B) Optogenetic neural stimulation of cardiac tissue via Channelrhodopsin-2 (ChR2), selectively expressed only in the neurons. Co-cultures of neurons and myocytes (loaded with dye Di-4-ANBDQBS spectrally compatible with ChR2). Optical stimulation (470 nm) was provided at pulse lengths of 3 s, at 0.5 Hz, using irradiance of 0.5–1 mW/mm^2^. (Bi) Post-processed traces using custom-written MATLAB software. Traces showing baseline no activity and followed by long light pulse stimulation, action potentials are evoked indirectly in the myocytes via the ChR2-light-sensitized neurons. Blue is the trace after baseline subtraction after median filtering, red indicates detected spike times, black is an indicator of when light is present (black down = light off). (Bii) The number of neurons innervating the myocytes affects the firing frequency of myocytes in cultures with different neuron to myocyte ratios (0 = myocytes/controls, 1 × 10^−5^ = 1:100,000, 0.01 = 1:100, 0.05 = 1:20 and 0.2 = 1:5); the number of experiments (n) for each group were 14, 4, 23, 28, and 12 and confidence values (p) (against null hypothesis of zero effect; Wilcoxon signed rank test) were 0.7131, 0.6250, 0.1091, 0.0000, and 0.0352. We observe a dose-dependent effect (i.e. the greater the number of neurons innervating the myocytes, the greater the effect, with values greater than 0 indicating an increase in beat rate). (C) Photo-uncaging of nicotine using a flash of blue light may lead to the release of noradrenaline by the sympathetic neurons resulting in increase in myocyte beat rate. Panel A was created with Servier Medical Art according to a Creative Commons Attribution 3.0 Unported License guidelines 3.0. See also Figure S4 for immunohistochemistry; see also Figures S15–S17. SN: sympathetic neuron; N: neuron, CM: cardiomyocyte, HT: high throughput.

Co-cultures were created by varying the neuron concentration cultured together on top of a monolayer of myocytes. Myocytes were plated in 96 well plates at plating density of 140,000 myocytes/well. The stellate sympathetic neurons were infected with hChR2-eYFP. Schematic representation of the co-cultures with different neuron dosing regimes (neuron-myocyte ratios in 1:5, 1:20, 1:100, 1:100,000) are shown in Figure 5A. Immunohistochemistry was used to confirm the presence of sympathetic neurons (using TH antibody) in the different neuron-myocyte co-culture ratio combinations (Figure S4). High-throughput fluorescence imaging methods (Klimas et al., 2020) of co-cultures also allowed the study of the effects of sympathetic cardiac stellate neurons on cardiac activity in well-connected quiescent cardiac cultures using optical mapping (cultures loaded with the near-infrared voltage-sensitive dye Di-4-ANBDQBS). Co-cultures were found to be constitutively more active than monocultures of myocytes (Figure S14) [Fishers exact test (two-sided), p = 0.0046 statistically significant, n = 6 myocytes and n = 24 for co-cultures].

Optogenetic neural stimulation of cardiac tissue via channelrhodopsin-2 (ChR2), selectively expressed only in the neurons, was performed using a light stimulation protocol schematically represented above plot 5 B(i) in Figure 5. Co-cultures of neurons and myocytes were loaded with dye Di-4-ANBDQBS, which is spectrally compatible with ChR2 (Klimas et al., 2020). Optical stimulation (470 nm) was provided at pulse lengths of 3 s, at 0.5 Hz, using irradiance of 0.5–1 mW/mm^2^. Long light pulse stimulation results in action potentials evoked indirectly in the myocytes by ChR2-expressing neurons (Figures 5B(i) and S15E). Cardiac response to light stimulation of ChR2-expressing neurons shows a dose-dependent effect (Figure 5B(ii)), where cultures with higher neuron concentrations generate more cardiac activity with the same light stimulus.

The normalized firing score is given as:

$$\frac{\frac{Ns}{Ps}-\frac{Nns}{Pns}}{\frac{Ns}{Ps}+\frac{Nns}{Pns}}$$

where Ns and Nns give the number of detected spikes during periods of stimulation and no stimulation, respectively. Ps and Pns give the fraction of the recording that is stimulated and not stimulated, respectively. This score lies between −1 (all spikes occurring during the nonstimulated period) and 1 (all spikes occurring during the stimulated period).

We also assessed the co-culture dynamics using an optochemical approach where caged nicotine was added to each well (Figure 5C), and nicotine is released with light stimulation (see also Figure S16). Although we observed cases where low-density co-cultures responded to nicotine uncaging (12/18 responders), either by increasing beat rate or by inducing bursting behavior in the cardiac monolayer, these responses occurred after a long delay, which raised the possibility that the effects may be due to chance. Longer recording times both pre- and post-light stimulation are required to confirm the efficacy of this method.

Standard (global) nicotine treatment of co-cultures to drive sympathetic neurons offers very little spatiotemporal control over the experiments (Figure S17). Optochemical methods to cause uncaging of nicotine to stimulate neurons, which in turn stimulate myocytes, can be achieved (Figures 5 C and S16). The timing of chemical release to stimulate neurons in culture to alter myocyte response (rate) can be controlled with some precision using this approach. At the same time, optogenetic stimulation of stellate sympathetic neurons offers far superior precise spatiotemporal control of neuron behavior and their effects on myocytes (Figures 5Bi and S15).

## Discussion

Emerging evidence supports the potential of neuromodulation therapy in clinical management and prevention of lethal arrhythmias (Meng et al., 2018), but direct neural-cardiac interactions (at the cell level) are still understudied due to lack of specific tools with high spatiotemporal resolution. We demonstrate that interesting topics such as the effect of neural density on the electrophysiological properties of a cardiac syncytium can be studied at the multicellular level in a high-throughput manner using all-optical techniques. We report the rather unexpected finding that neuronal activation protects from developing activation wave breaks, regarded as an *in vitro* marker for arrhythmogenic behavior. Our work leveraged the use of dye-free mapping of activation wave fronts over a large field of view. Here we highlight (i) a methodology to study neuron-cardiac interactions at the multicellular/tissue level; (ii) findings on the relationship between neuron presence and macroscopic wave properties; (iii) and finally the relationship between neuron density in co-culture and cardiac firing rates. Photo-uncaging of nicotine or optogenetic neural stimulation are used here in conjunction with optical imaging of cardiomyocyte contractile and electrical activity to illustrate the power of such interrogations. We designed a simplified *in vitro* model of neurally modulated arrhythmogenesis by co-culturing stellate sympathetic neurons with confluent monolayers of myocytes and optically measured the effects of these neurons on cardiac wave speeds. We demonstrate that physical (Figure 1) and functional connections (Figures 2, 3, 4, and 5) are formed between cardiac sympathetic stellate nerve cells and cardiomyocytes as previously reported in isolated cell preparations (Himel et al., 2012; Prando et al., 2018; Tao et al., 2011).

Using optogenetic stimulation of catecholaminergic neurons (targeted via a TH-promoter) in transgenic mouse hearts, Wengrowski et al. (2015) showed immediate increase in heart rate and contractility. The basis of this interaction is the existence of specialized junctional sites between neurons and myocytes shown by Shcherbakova et al. (2007) and later Prando et al. (2018), who used coupled myocyte pairs to demonstrate that neurons raise intracellular cAMP only in directly contacted myocytes. In addition, there is a wealth of evidence that cardiac innervation is tightly linked to cardiac development and function. For example; nerve growth factor is required for sympathetic axon growth and innervation (Ieda et al., 2004; Kuruvilla et al., 2004; Lockhart et al., 2000) and SEMA3A expression is needed for sympathetic innervation patterning and appears to be critical for heart control (Ieda et al., 2007). Although these studies highlight the importance of local neuron myocyte connections, they raise questions related to how the density and distribution of these connections impact macroscopic wave propagation at the tissue level.

Microscopy studies have demonstrated that the density of cardiac innervation is normally very high (Freeman et al., 2014), with neuronal processes being close to almost every cardiomyocyte in intact myocardium. Neuron density can, however, dramatically change in response to disease states. For example, there is loss and gain of sympathetic axons in the border zone of a chronic myocardial infarction (Cao et al., 2000; Freeman et al., 2014; Zhou et al., 2004). The functional consequences of these variations remain unclear.

Although our experiments demonstrate that innervation results in functional changes in CV and changes the spatial organization of cardiac waves *in vitro*, the mechanisms responsible for these changes are unknown. Fibroblast concentration, gap junction density, and ion channel expression are known modulators of conduction velocity. Our immunofluorescence studies indicate low vimentin expression levels in the cultures, indicating low fibroblast proliferation (Figure S3); however, preliminary studies on gap junction protein Cx43 levels found that this protein was elevated in co-cultures relative to cardiac monocultures (Figure S12). In addition, we performed a label-free quantitative proteomics analysis on cultures (see Tables S1, S2, S5, and S6); protein ratios (co-cultures/myocytes) were calculated from SINQ intensities (see Tables S5 and S6) for all quantified protein hits; and the regulation of randomly chosen proteins fibronectin and vimentin observed in proteomics were confirmed by western blots in 3 independent experiments (see Figure S11). We found changes in pathways regulating gap junction protein expression along with numerous changes in pathways associated with metabolism and development (Figures S8 and S10). The results of our proteomics screen is consistent with other studies that link innervation to developmental processes (Atkins et al., 1997; Larsen et al., 2016; Ogawa et al., 1992; Shcherbakova et al., 2007; Takeuchi et al., 2011). Shortly after birth, cardiomyocyte hyperplasia decreases and CV increases (Ogawa et al., 1992), along with increased β-adrenoreceptor density on cardiomyocytes and higher levels of catecholamines in the circulation (Claycomb, 1976). Recent studies (Kreipke and Birren, 2015) have demonstrated that having sympathetic neurons present in *in-vitro* cardiac cultures delays cardiomyocyte cell cycle withdrawal and transiently limits hypertrophy via a β-adrenergic signaling pathway, which suggests that sympathetic innervation can regulate cardiomyocyte numbers during the postnatal period. Developmental changes may occur in neurons as well: Oh et al. reported increased maturation of hiPSC-derived sympathetic neurons in their cardiac neuron co-culture system (Oh et al., 2016). Coppen et al. have suggested the existence of post-natal changes in connexin expression in the developing fetal heart (Coppen et al., 2003). There is also additional evidence that expression of different connexin isotypes varies not only within distinct compartments of the adult heart but also as a function of cardiac developmental stage (Giovannone et al., 2012). It is possible that neurons enhance maturity of cardiomyocytes and the CV increase seen in our experiments may be due to developmental changes in the cardiac myocytes and improved connectivity.

Furthermore, our tissue culture results may also be relevant to understanding the effects of nerve sprouting in scar tissue and in helping to resolve the apparently conflicting results summarized in the Introduction. Our observation that co-cultures display fewer wave breaks than monocultures, have increased CV, and higher levels of Cx43 offer indirect support for the protective role of neurons in intact tissues, particularly in cases such as the infarct border zone, which shows reduced function of gap junctions and slower conduction (Luke and Saffitz, 1991). At the same time, the effects of acute nicotinic stimulation of neurons in our co-culture system may be a model of proarrhythmogenicity in the hyperinnervated infarct border zone during a surge of the sympathetic drive.

Although conventional electrophysiology techniques allow for specific micro control of single cells or sparse cell cultures, the application of such techniques is technically challenging when studying two cell types grown in a syncytium (such as the spontaneously excitable cardiac tissue and neurons). Arthur Winfree (Winfree, 1987) hypothesized many years ago that the pattern of nervous system innervation could determine whether an arrhythmia could be instigated. Alterations in autonomic function occur in several interrelated cardiac conditions including sudden cardiac death, congestive heart failure, diabetic neuropathy, and myocardial ischemia (Vaseghi and Shivkumar, 2008). Neural modulation as a treatment for arrhythmias has been well established in certain diseases (such as long QT syndrome); however, in most other arrhythmias, it is still an open question and the subject of intense research (Herring et al., 2019). Ongoing research over the last five decades has highlighted the importance of communication between neural and cardiac tissues. The evidence that the role of excessive cardiac sympathetic activity can directly precipitate ventricular tachycardia has been provided by studies in patients and animal models with healed myocardial infarction (Billman, 2006; Janse et al., 1985; Jiang et al., 2008). However, the technical challenges of performing electrophysiological experiments motivated us to adopt an alternative, more “controlled” method to study the effects of neuron numbers on cardiac behavior, and these data are presented in Figure 5B where we opted for an optogenetic approach to control neurons and study the resulting cardiac behavior. We observe that increasing the number of neurons innervating the myocytes affects the firing frequency of myocytes in cultures. We report a dose-dependent effect (i.e. the greater the number of neurons innervating the myocytes, the greater the effect), where the highest effect on rate increase is observed in the 1:5 neuron-myocyte ratio cultures. Co-culture studies (Lockhart et al., 1997, 2000) focusing on myocyte maturation, nerve growth factor, and synapse formation between myocytes and sympathetic neurons have used similar physiological neuron-myocyte ratios (∼7500 neurons to ∼75,000 myocytes).

In terms of neurocardiac interactions, Lockhart et al. (2000) put the average process length per neuron in co-culture to be 1.8 ± 0.3 mm. We measured process length in SEM images and estimated the average process in our cultures to be 1,183 μm. Therefore, in 67% of cases each neuron will directly stimulate between 56 and 167 myocytes, with an average of 83 myocytes per neuron. This estimate is based on a two-dimensional model of neuron innervation and assumes that each point of contact between neuron and myocyte leads to a site of innervation.

The number of myocytes needed to initiate a wave of activity has been investigated using a variety of techniques and can be in the thousands in healthy, well connected tissue (Tveito and Lines, 2008; Xie et al., 2010; Zaglia et al., 2015). However, the minimum number of myocytes needed strongly depends on their connectivity, with simulations suggesting that this may be as low as 40 cells in poorly connected, fibrotic tissue. As the conduction velocity in our preparation (39 mm/s) is lower than those simulations (60 mm/s in fibrotic tissue simulations, Table 1 in Xie et al. (2010)), and since conduction velocity is a monotonic function of gap junction connectivity (Dhillon et al., 2013), these modeling studies provide a plausible mechanism for modulation of tissue level cardiac activity by a single neuron. The surprising neuron patch clamp pilot study result (Figure 4B) indicating that one neuron can potentially stimulate a connected monolayer of cardiac cells calls for further consideration for the role of neuron numbers and their potential impact during pathological conditions. We note in the experiments where 1:100,000 neuron/cardiomyocyte density was studied, the cardiac response to neuron stimulation was negligible; however, it is important to note that at these densities some of the measured wells likely had zero neurons (see Transparent Methods, Section 4). From our experiments and previously published literature, it seems plausible that dendrites of one neuron can stimulate (through NA release) a big group of myocytes, which in turn then go on to stimulate their neighbors and consequently, the whole dish.

Although experimental challenges still need to be overcome, dissecting mechanisms along the heart-brain axis has become more achievable with the introduction of innovative methods, imaging (Bruegmann et al., 2010; Prando et al., 2018; Sigalas et al., 2020; Vaseghi et al., 2012), and tissue engineering techniques (Burton et al., 2015; Klimas et al., 2016). In summary, we investigated the effect of sympathetic innervation on the activation dynamics of a cardiac cell monolayer innervated *in vitro* by co-culture with stellate ganglia neurons. We report the rather unexpected finding that neuronal activation protects from developing activation wave breaks, regarded as an *in vitro* marker for arrhythmogenic behavior. Does the number of neurons innervating cardiac tissue matter? Our data suggest that the greater the number of neurons innervating the myocytes, the greater the cardiac effect observed. The utility and scope of our macroscopic co-culture model offers even greater potential. In addition to using dye-free approaches (Burton et al., 2015) to measure pattern formation and conduction velocity, all-optical electrophysiology allows for high-resolution, high-throughput fluorescent interrogation of neural influence on cardiac monolayers using optochemical and optogenetic stimulation (Figure 5) (Klimas et al., 2016), (Klimas et al., 2020). We have also extended this line of investigation from primary neonatal rat cells to human iPSC-derived peripheral neuron co-cultures as a proof of concept study (Axiogenesis [now Ncardia], Figure S18). Thus, the methods described here provide approaches that could broaden our insight into fundamental human disease mechanisms. The use of *in vitro* techniques in pharmacological assays and profiling is growing in its popularity in the drug discovery process (Bowes et al., 2012). Our experimental model in conjunction with recently developed imaging platforms can be applied to improve the efficacy of preclinical drug toxicity and discovery studies. More structural and mechanistic knowledge on the sympathetic neuron numbers and patterns in the heart could offer a new step toward potential therapies for lethal arrhythmias.

### Limitations of the Study

Although dye-free imaging offers long-term, non-contact precision control of wave properties that pharmacological and electrical methods lack, the interpretation of complex spiral wave patterns may be hindered by similarities in the optical signals from excitation and relaxation waves (Sigalas et al., 2020). Low-density neuron co-culture dishes offered interesting observations that may be of clinical relevance; however, the technical challenges of ensuring low numbers of neurons that are present and functional in these co-cultures currently highlight a limitation of this approach. Furthermore, the experiments in the present study did not control for organizational variations inherent within monolayer cultures. Although the techniques utilized here at the macroscopic level have clear advantages over the use of single-cells and monocultures, correlation of events observed at the neuron-cardiac junction in co-culture monolayers with *in vivo* activity will require further validation in intact tissue. In addition, current techniques for generating monolayer cultures rely on the harvesting of cardiomyocytes from neonatal animals. It is important to recognize that such neonatal cells exhibit a different morphology and phenotype compared with mature cells, further highlighting the requirement for *in vivo* validation at the level of the intact, mature heart. Future efforts can adapt these approaches to optimized human stem-cell-derived cardiomyocyte tissue constructs and stem-cell-derived autonomic neurons for better relevance to human physiology.

### Resource Availability

#### Lead Contact

Further information and requests for resources should be directed to and will be fulfilled by the Lead Contact, Rebecca Burton (Rebecca.burton@pharm.ox.ac.uk).

#### Materials Availability

This study did not generate new unique reagents.

#### Data and Code Availability

No new specialized code was used.

Proteomics Data: the mass spectrometry proteomics data have been deposited to the ProteomeXchange Consortium via the PRIDE (Perez-Riverol et al., 2019) partner repository with the dataset identifier PXD019908 and 10.6019/PXD019908.

The imaging datasets supporting the current study have not been deposited in a public repository because of the large nature of the files (∼1 Terabyte data) but are available from the corresponding author on request.

## Methods

All methods can be found in the accompanying Transparent Methods supplemental file.

## Supplemental Information

Document S1. Transparent Methods and Figures S1–S18

Table S1. Protein Identification in Myocyte and Neuron-Myocyte Co-cultures by Liquid Chromatography-Tandem Mass Spectrometry (LC-MS/MS) Analysis and Database Searching, Related to Figures 2 and S7 (Sample SD22) Protein extracts were processed by in-gel trypsin digestion and LC-MS/MS analysis of the tryptic peptides. LC-MS/MS raw data files were converted to Mascot generic files (.mgf) and proteins identified by database searching of the Uniprot-Swissprot database (taxonomy restriction: rat) using the MASCOT search algorithm. A target-decoy search strategy was employed, and data are displayed at a 1% false discovery rate (FDR).

Table S2. Protein Identification in Myocyte and Neuron-Myocyte Co-cultures by Liquid Chromatography-Tandem Mass Spectrometry (LC-MS/MS) Analysis and Database Searching, Related to Figures 2 and S9 (Sample SD5) Protein extracts were processed by in-gel trypsin digestion and LC-MS/MS analysis of the tryptic peptides. LC-MS/MS raw data files were converted to Mascot generic files (.mgf) and proteins identified by database searching of the Uniprot-Swissprot database (taxonomy restriction: rat) using the MASCOT search algorithm. A target-decoy search strategy was employed, and data are displayed at a 1% false discovery rate (FDR).

Table S3. Gene Ontology and Expressence Analysis of Regulated Protein Hits (≥2-Fold up- or Downregulation) in the Comparison of Primary Myocyte and Neuron-Myocyte Co-cultures, Related to Figures 2 and S8 (Sample SD22) Analysis was carried out with the Biological Network Gene Ontology tool (BiNGO). -tab ST3A) 22SDallclusters-BP—this contains the Bingo hypergeometric tests against the Biological Process (BP) gene ontology for 22SD experiment; -tab ST3B) 22SDallcluster-CC—this contains the Bingo hypergeometric tests against the cellular component (CC) gene ontology for 22SD experiment; -tab ST3C) 22SDallcluster-MF—this contains the Bingo hypergeometric tests against the molecular function (CC) gene ontology for 22SD experiment.

Table S4. Gene Ontology and Expressence Analysis of Regulated Protein Hits (≥2-Fold up- or Downregulation) in the Comparison of Primary Myocyte and Neuron-Myocyte Co-cultures, Related to Figures 2 and S10 (Sample SD5) Analysis was carried out with the Biological Network Gene Ontology tool (BiNGO). -tab ST4A) SDCN5allclusters-BP—this contains the Bingo hypergeometric tests against the Biological Process (BP) gene ontology for SDCN5 experiment; -tab ST4B) SDCN5allclusters-CC—this contains the Bingo hypergeometric tests against the cellular component (CC) gene ontology for SDCN5 experiment; -tab ST4C) SDCN5allclusters-MF—this contains the Bingo hypergeometric tests against the molecular function (MF) gene ontology for SDCN5 experiment.

Table S5. Spectral Index Quantitation (SINQ) of Proteins Identified in Primary Myocyte and Neuron-Myocyte Co-culture from Sprague Dawley Neonatal Rats, Related to Figure 2 (Experiment SD22) The analysis was performed with the integrated SINQ algorithm within the central proteomics facilities pipeline. Maximum protein group q-value: 0.01; minimum of 2 unique peptide sequences per protein hit in at least one search.

Table S6. Spectral Index Quantitation (SINQ) of Proteins Identified in Primary Myocyte and Neuron-Myocyte Co-culture from Sprague Dawley Neonatal Rats, Related to Figure 2 (Experiment SD5) The analysis was performed with the integrated SINQ algorithm within the central proteomics facilities pipeline. Maximum protein group q-value: 0.01; minimum of 2 unique peptide sequences per protein hit in at least one search.
