# Supplementary material for: Optical Interrogation of Sympathetic Neuronal Effects on Macroscopic Cardiomyocyte Network Dynamics
Source: iScience. 2020 Jul 1;23(7):101334. doi: 10.1016/j.isci.2020.101334 (PMC7363704; doi:10.1016/j.isci.2020.101334)

iScience, Volume *23*

Supplemental Information

Optical Interrogation of Sympathetic Neuronal Effects on Macroscopic Cardiomyocyte Network Dynamics

Rebecca-Ann B. Burton, Jakub Tomek, Christina M. Ambrosi, Hege E. Larsen, Amy R. Sharkey, Rebecca A. Capel, Alexander D. Corbett, Samuel Bilton, Aleksandra Klimas, Guy Stephens, Maegan Cremer, Samuel J. Bose, Dan Li, Giuseppe Gallone, Neil Herring, Edward O. Mann, Abhinav Kumar, Holger Kramer, Emilia Entcheva, David J. Paterson, and Gil Bub

TRANSPARENT METHODS:

All UK experiments were performed in accordance to UK Home Office Animals Scientific Procedures Act (1986). All experiments performed in the US were per an approved Stony Brook University IACUC protocol.

1] Cell Cultures:

a) Spontaneously active cell culture (Oxford culture): Here we use a neonatal ventricular cardiac monolayer cell culture model that spontaneously displays a wide range of behaviors (Burton *et al.*, 2015) to investigate how neurons modulate pacemaking and reentrant activity. We measured activity (i) using a macroscopic dye free optical mapping imaging modality, (ii) patch clamp electrophysiology coupled with video microscopy and (iii) microscopic optical mapping. Hearts were isolated from neonatal SD rat pups (P1-P3), killed by Schedule 1 in accordance to UK Home Office Animals Scientific Procedures Act (1986). Ventricular myocytes were enzymatically isolated by a series of enzymatic digestions in trypsin (1mg/mL, Sigma Aldrich, UK) followed by collagenase (1mg/mL, Sigma Aldrich, UK) and triturated to achieve a suspension of cardiomyocytes. The isolated cells were then pre-plated in an incubator (37°C, 5% CO2) for an hour to allow most fibroblasts to settle at the bottom of the dish. The ventricular myocytes in the supernatant were then carefully removed from the dish and a cell count performed using a haemocytometer and trypan blue. The myocytes were plated on 35 mm poly-lysine coated petri-dishes (Bio coat Poly-D-Lysine 35mm petri-plates, Corning, UK) at a density of 750,000 cells (per 35 mm petri-dish) in plating medium (85% DMEM, 17% M199, 10% Horse serum, 5% FBS and 1% penicillin/streptomycin, all from Sigma Aldrich). 24 hours later the cardiac sympathetic stellate neurons were isolated from litter mates as described previously with some modifications (He and Baas, 2003, Li *et al.*, 2012). Briefly, following microscopic dissection of the sympathetic stellate ganglia, enzymatic digestion in trypsin 1mg/mL (Worthington, USA) and collagenase type-4 1 mg/mL (Worthington, USA), cells were dissociated by sequential mechanical trituration using fine (fire-polished) glass pipettes. Neurons were pre-plated for 1 hour (to eliminate fibroblast and Schwann cells), counted and plated in at varying neuron to myocyte ratios. Co-cultures were created by plating neurons on top of the cardiac monolayers. Co-cultures were maintained in media supplemented with nerve growth factor (50 ng/mL, NGF Millipore), which promotes neuron development. The same high serum level media (maintaining the original serum concentrations of 10% horse serum and 5% FBS), was refreshed every other day, which supports the spontaneously active cardiac monolayers. The pre-plating steps and serum levels differ from the SBU cultures described in 1(b), which are the key differentiating steps and between these cultures that are spontaneously active.

<u>Stimulation of cardiac sympathetic stellate neurons with nicotine</u>: To measure the effects of neuron stimulation on myocyte beat rate, 10 μM nicotine ([−] nicotine hydrogen tartrate salt; Sigma-Aldrich) was used. Washout was performed with pre-warmed media.

b) **Neonatal rat ventricular cardiomyocyte Culture (Stony Brook University culture-SBU)**: Neonatal (2–3-day old) Sprague–Dawley rats were killed by Schedule 1 and ventricular tissue was removed as per an approved Stony Brook University IACUC protocol (New York, USA). The ventricular tissue was digested in trypsin made up in in Hanks' Balanced Salt Solution (1 mg/mL, US Biochemical, Cleveland, OH) overnight at 4°C. The tissue was serially digested using 1 mg/mL collagenase (Worthington Biomedical, Lakewood, NJ) in HBSS at 37 °C and pipetted into conical tubes and placed on ice. After centrifugation, cells were re-suspended in culture medium M199 (GIBCO) supplemented

with 12 μM L-glutamine (GIBCO), 0.05 μg/mL penicillin-streptomycin (Mediatech Cellgro, Kansas City, MO), 0.2 μg/mL vitamin B12 (Sigma-Aldrich, St. Louis, MO), 10 mM HEPES (GIBCO), 3.5 mg/mL D-(+)-glucose (Sigma-Aldrich) and 10% fetal bovine serum, FBS (GIBCO). Fibroblasts were removed via a two-step pre-plating process, where the cell suspension was plated in a flask and incubated (37 °C, 5% CO2) for 45–60 min and switched to a new flask and the incubation repeated. Cardiomyocytes were then counted using a hemocytometer before plating in glass-bottom 96-well plates (*In Vitro* Scientific). 24 hours later the cardiac sympathetic stellate neurons were isolated from litter mates and co-cultured with the cardiomyocytes as described above in Cell Culture Methods. Low serum (2%) maintenance media was used from day 2.

96 well microscopic optical recording of SBU co-cultures with voltage-sensitive dye using high throughput fluorescent methods: Isolated neonatal cardiac cells were plated on fibronectin coated 96-well glass-bottom plates (In Vitro Scientific, P96-1-N). After 24 hours, stellate ganglia were isolated from litter mate pups (P3) and neurons isolated as described above, and plated on top of the myocytes. We performed optical recording of membrane voltage using the voltage-sensitive dye Di-4-ANBDQBS (from Leslie Loew, University of Connecticut), in normal Tyrode solution (4-5 days after cell plating). We compared a high dose versus low dose myocyte neuron co-culture combination (i.e. 1:5 (1 neuron stimulating 5 myocytes) and 1:100,000 (1 neuron stimulating 100,000 myocytes)) to determine the effect of neuron density on myocyte activity. Neurons were stimulated using 100 μM caged nicotine (RuBi-Nicotine, Cat No. 3855, Tocris). Measurements were carried out at room temperature as the multi-well plate setup did not accommodate a stage top incubator. We only analysed data from wells where the tissue was quiescent or beating with a rate lower than 1 Hz before nicotine stimulation: in particular recordings from monolayers that displayed bursting dynamics before nicotine stimulation were not used. Three of the wells displayed bursting dynamics before nicotine stimulation and were excluded from subsequent analysis.

Drugs: Stimulation of cardiac sympathetic stellate neurons with nicotine: To measure the effects of neuron stimulation on myocyte beat rate, 10 μM nicotine ([−] nicotine hydrogen tartrate salt; Sigma-Aldrich) was used. Washout was performed with pre-warmed media. In case of caged nicotine experiments, caged nicotine known as RuBiNic (RuBi-Nicotine, Cat No. 3855, Tocris) has been used in neurotransmission studies (Filevich *et al.*, 2010b). Uncaging can be extremely rapid, controlled in time or space and quantitatively controlled and repeated. We tested the following neuron-myocyte densities: 1:5, 1:20 and 1:100. We employed caged nicotine at 100 μM as the actual amount of uncaged drug is usually extremely small based on details in (Macgregor *et al.*, 2007).

2] Dye-free measurement of wave dynamics (Oxford dye-free macroscopic imaging): Our experimental goal was to determine how the addition of neurons modulates activation patterns in cardiac culture under a wide range of initial conditions. We found that cultures plated on poly-D-lysine coated plastic petri dishes and continuously maintained high serum conditions (10% Horse serum, 5% FBS) resulted in isotropic cultures that spontaneously display a wide range of excitation patterns (called here Oxford cultures).

Experimental conditions between research groups differ, but typically cultures by other groups are prepared by plating tissue on fibronectin coated glass coverslips, use low serum conditions after two days in culture, and perform imaging experiments after transferring dishes to a chamber in standard Tyrode solution in normal atmosphere. However, in our

hands cultures prepared in these conditions often require pacing to induce activity, which was not compatible with our experimental aim of finding how the addition of neurons modulate spontaneous cardiac activity.

We used dye-free imaging techniques (Hwang *et al.*, 2005) with modifications as described in (Burton *et al.*, 2015) to image the spontaneously active confluent monolayers (Oxford cultures). Here, we employed an Olympus MVX10 Macroscope and Andor Neo sCMOS camera to record wave patterns and beat rate (from day 3 onwards). Experiments were carried out in an Okolab (Indigo Scientific, UK) stage incubation chamber controlled for heat (32 - 37˚C), CO2 (5%) and humidity. Culture plates were allowed to equilibrate in these conditions for 20 minutes before commencing recordings. From these we were able to observe the propagation of cardiac waves across the plates by detecting the minute contractile motions of the myocytes. Frame rates were captured between at 50 fps. The software for displaying the camera output in real time, saving and analyzing data were written in a combination of Java and Python, and is directly available from the authors. For detailed description of the methods see (Burton *et al.*, 2015). To measure the effects of neuron stimulation on myocyte beat rate, 10 µM nicotine ([−] nicotine hydrogen tartrate salt; Sigma-Aldrich) was used to trigger depolarisations. Washout was performed with pre-warmed media (similar to the methods described by (Furshpan *et al.*, 1976)).

Imaging setup: We developed a dye free imaging system similar to that described in (Hwang *et al.*, 2005), where off-axis oblique illumination is used to generate high contrast images that can be analysed to extract wave activity. We employ an Olympus MVX10 macroscope to relay a 1x image to a Neo sCMOS camera running at 50 frames/second. The high resolution of the camera allows visualization of the contraction of the tissue at the cell level, while still giving a relatively large 16 x 16 mm field of view. High resolution frames are processed so each displayed frame is generated by subtracting a frame captured at t-300 ms from the current frame and displaying the absolute value of each pixel. Intensity vs. time traces are obtained by 5x5 pixels in the image. The intensity vs. time traces typically have a double hump morphology which is due to contraction (first hump) followed by relaxation (second hump) of the tissue (Figure S 5 bottom right trace).

Wave speed measurement: We employed a semi-automated approach for finding cardiac waves in optical mapping recordings and measuring their speed of propagation. Data files are preprocessed by first extracting motion transients by finding the absolute value of the intensity differences for every pixel over a 6-frame rolling window (see Burton *et al.*, 2015), and performing a 8x8 spatial binning operation to give a 256x256 pixel stack of frames. Wavefronts are visually identified by the operator, who draws a path perpendicular to the wavefront along the propagation direction. The software then calculates the 50% maximum intensity crossing time for two rectangular (10 x 20 pixel) ROIs positioned at the ends of the drawn path. A conduction velocity measurement is calculated based on the path length and ROI threshold crossing times. Several (an average 14) measurements are made at different locations and times for each record, and the 90th percentile of these is saved as a representative conduction velocity for that experiment. We compare conduction velocities from 22 co-culture and 19 myocyte only cultures. Normal distribution of the data was confirmed using the Kolmogorov-Smirnov test and data were compared using unpaired, two-tailed t-test ($P = 0.026$, data shown as mean ± SD). Our automated conduction velocity measurement system yields lower values than measurements of planar waves in paced tissue, which may be due to the inclusion of slowly moving highly curved wavefronts in the calculations (e.g. wave fronts near the core of a spiral wave). We note that the maximal

observed wave velocity in our preparations was 133 mm/second, which is similar to planar wave conduction velocities in isotropic tissue reported by other groups.

3] Simultaneous patch clamp electrophysiology-video recording: Current clamp was used to record electrophysiological traces from neurons, while video recording of cardiac behaviour was performed simultaneously using a CCD camera. The signal was recorded and analysed using custom-made procedures in Igor Pro (Wavemetrics). Image series were after processing the images so that the value of each pixel p at frame t, denoted p(t), is replaced by the absolute value of p(t) - p(t-5).

Current clamp recordings were performed with a Multiclamp 700B amplifier (Molecular Devices). Borosilicate glass pipettes were filled with an internal solution containing (in mM): 110 potassium-gluconate, 40 HEPES, 2 ATP-Mg, 0.3 GTP and 4 NaCl (pH 7.2–7.3; osmolarity 270–285 mOsmol). Standard artificial cerebrospinal fluid (ACSF) bubbled with 5% CO2 was used as the bath solution, containing (in mM): 126 NaCl, 3 KCl, 1.25 NaH2PO4, 2 MgSO4, 2 CaCl2, 26 NaHCO3, and 10 glucose, pH 7.2–7.4. All recordings were conducted at 37°C. Square pulses of current or current steps were administered to depolarise the neurons and elicit action potential firing. Data were low-pass filtered at 2 kHz and acquired at 5 kHz.

4] Assessment of cardiac activity in co-cultures with low neuron density using high throughput fluorescence methods on SBU culture:
Caged-Nicotine:
Electrophysiological experiments studying the effects of noradrenaline release by sympathetic neurons on cardiac behaviour employ methods that flow on drug and monitor voltage or calcium changes either via the patch pipette or beat rate changes using video microscopy. We first tested the co-cultures using conventional pharmacological approaches (with Sigma 50 µM nicotine stimulation), to demonstrate the expected results of noradrenaline release by stellate sympathetic cardiac neurons on myocytes. Conventional methods such as the aforementioned do not offer any temporal or spatial control. Such a technique does not bode well with high-throughput imaging methods as the drug effect and the imaging timings cannot be precisely coordinated.

Due to technical challenges in co-culture patch clamp method, we performed a pilot study where a single patched neuron, driven to rapidly fire by injection of current, modulated the activity of a confluent monolayer. While this pilot experiment suggested that a single rapidly firing neuron can drive the behavior of a macroscopic cardiac monolayer, this protocol cannot rule out network effects from unpatched neurons. However, performing patch experiments on co-cultures with very few neurons (e.g. theoretically calculated one neuron per monolayer) was not practical as it proved to be technically difficult to locate a neuron amongst thousands of cardiomyocytes within an acceptable time frame. We therefore opted to investigate the activity co-cultures with low neuron numbers (calculated for 1 neuron:100,000 myocytes) using opto-chemical stimulation and a previously published high throughput fluorescent imaging technique (Klimas *et al.*, 2016). Since the low number of neurons increased variability, experiments were performed using multi-well plates to obtain sufficient statistical power. The use of multi-well plates necessitated the use of a modified experimental setup that differed from the one used to image activity in conventional petri plates as follows.

Due to temporal limitations of drug administration and effects, an alternative technique suitable to high-throughput measurements employing caged compounds was employed. Caged compounds are light-sensitive probes that functionally encapsulate molecules first introduced in the 1970's. These molecules are in an inactive form and upon irradiation, liberate the trapped molecule allowing targeted perturbation of physiological processes (Ellis-Davies, 2007). Caged nicotine known as RuBiNic (RuBi-Nicotine, Cat No. 3855, Tocris) has been used in neurotransmission studies (Filevich *et al.*, 2010a). Uncaging can be extremely rapid, controlled in time or space and quantitatively controlled and repeated. We tested the following neuron-myocyte densities: 1:5, 1:20 and 1:100,000 (see Figure S 4 for immuno-histochemistry staining of neurons at different neuron-myocyte co-culture ratios).

Opto-chemical neuronal stimulation and the effect of neurotransmitter release on cardiomyocytes:

Neonatal rat ventricular cardiomyocyte/co-cultures (SBU culture) were employed (see Supplementary Methods point 1b). Importantly, cardiac/co-cultures were maintained in low serum (2%) maintenance media from day 2. Optical recording of membrane voltage, Vm, was performed using the synthetic voltage-sensitive dye Di-4-ANBDQBS. 17.5 mM of stock solution in pure ethanol is diluted to 35 µM Tyrode's solution. Cells are stained for 6 min in dye solution followed by a 6 min wash in fresh Tyrode's. Finally, the wash solution is replaced with fresh Tyrode's. SBU fluorescence imaging was performed at 200 fps with 4 × 4 binning using NIS-Elements AR (Nikon Instruments; Melville, NY). RuBi-Nicotine uncaging was elicited by a 150 ms, 1200 mA flash (470nm).

Neurons were stimulated using 100 µM caged nicotine (Tocris, USA)(Filevich *et al.*, 2010a) instead of free nicotine in solution in order to minimize the time between sequential measurements in multiple wells. The concentration of free nicotine following photorelease in these experiments was less than the concentration of the caged nicotine used (100 µM) as photorelease efficiency is poor at physiologically tolerated light levels. We tested the effects of caged nicotine between 10-100 µM and found that 100 µM resulted in neuronal driven cardiac responses similar to those evoked by the pure nicotine compound. We note that low photorelease efficiencies have been reported with similar compounds: Macgregor et al 2007 report a 2% efficiency of photorelease with caged NAADP (Macgregor *et al.*, 2007).

Neuronal optogenetics actuation:

The geometry of the multi-well plate (deep well relative to the bottom surface area) is not compatible with the oblique illumination method used for dye-free imaging used in larger plates due to unwanted light reflections of the sides of the well. In addition, the advantages associated with dye-free imaging (long term recording with low phototoxicity) are less relevant for these experiments as activity in different wells are measured in rapid succession for relatively short time periods. We therefore opted to measure cardiac activity using voltage-sensitive dye Di-4-ANBDQBS (10 µM, supplied by Dr Leslie Loew, University of Connecticut). A standard epi-fluorescence configuration was used (530 nm excitation light, with a 20x objective (20 x Nikon CFO Super Plan Fluor)).

The short time available for each recording and the microscopic field of view precludes measurement of CV and relatively subtle changes in patterns of activation. We therefore use experimental conditions that allow for an unambiguous assessment of the effects of nicotine on cardiac dynamics. For cell plating, 96-well glass bottom dishes (In Vitro Scientific) were coated with 50 µg/mL fibronectin (diluted in PBS), and incubated at 37 °C for at least 2 h before cell plating. Cells are plated in 10% FBS M199 media; on day 2, the media was

replaced with 2% FBS M199 until the day of experiments and imaged at room temperature, which favors quiescent or slowly beating cultures (Klimas *et al.*, 2016). The co-culture in each well can then be labeled as responsive or un-responsive to nicotine based on the induction of activity in normally quiescent cultures or a marked increase in beat rate in slowly beating cultures. Wells that display rapid or irregular activity before the addition of nicotine are excluded from subsequent analysis.

Each well is plated with 140,000 cardiomyocytes and seeded with neurons at a concentration of 1 neuron per 100,000 myocytes (theoretical aim). At this plating density, Poisson statistics predict that 24.6% of plates will have zero neurons, 34.5% will have one neuron, and 40.9% will have more than one neuron. The number of wells that respond to nicotine is used to determine the number of neurons needed to induce activity in a connected monolayer.

Optogenetic modification of neurons: Adenoviral vectors containing the transgene for hChR2(H134R)-eYFP were prepared in collaboration with the Stony Brook University Stem Cell Centre based on the expression cassette of the plasmid pcDNA3.1/hChR2(H134R)-eYFP (#20940; Addgene) (Ambrosi and Entcheva, 2014). Neurons were infected using Ambrosi's method (Ambrosi and Entcheva, 2014) with an optimised dose of adenovirus (multiplicity of infection (MOI) 2000) at 37°C for 2 h. Neuron expressing ChR2 was confirmed by eYFP reporter visualization. Following infection, fresh culture medium was added with a medium change at 12 h and then every 48 h with functional measurements performed on the sample from day 5 onwards (similar to studies by (Shcherbakova *et al.*, 2007, Larsen *et al.*, 2016). Optical recording of membrane voltage, Vm, was performed using the synthetic voltage-sensitive dye Di-4-ANBDQBS, spectrally compatible with ChR2. The light intensity threshold for ChR2 activation was obtained from previous studies(Ambrosi *et al.*, 2015). For Optogenetic experiments: Optical stimulation (470 nm) was provided at pulse lengths of 1 to 5 sec, at 0.5 Hz, using irradiances of 0.5–1 mW/mm. Whilst a wide range of pulse durations were tried, most the data analysed are in the 1-5 sec long pulses, Figure 5 B(i) is 3 sec per pulse.

(i) Beat rate changes in response to neuronal ChR2 stimulation: Figure 5 A illustrates the co-culture methods and high throughput all-optical interrogation approach. High throughput fluorescent imaging protocols of "optoelectrical" (via ChR2, Bi) vs "optochemical" (via Caged Nicotine, C) neural stimulation. Rat cardiac sympathetic stellate neurons are optogenetically transformed to respond to light activation, this promotes action potential firing in myocytes (Fig 5 B(i)). ChR2 expression by eYFP reporter (green) at four different neuronal plating densities 1:5, 1:20, 1:100 and 1:100,000 were studied. Optogenetic stimulation via ChR2: (Figure S 15 C) in the myocyte only culture shows no beat rate response, whereas in the co-culture (Figure S 15 D), we observe a beat rate response during the periods of light stimulation. Figure S 15 E, post processed traces using using custom-written Matlab software, where blue is the median filtering trace, red indicates detected spike times, black is an indicator of when light is present (black up=light on, 3 sec pulse).

Optochemical stimulation of neurons with nicotine (Figure S 16 B (ii): pre-uncaging, B (iii): post-uncaging, B (iv): returning to baseline after several minutes of uncaging) and optoelectrical stimulation of neurons via ChR2 (Figure S 15 E), demonstrate functional coupling between neurons and myocytes in co-cultures. We additionally test this coupling by traditional pharmacological antagonists (nicotine, Figure S 17). Genetically modified sympathetic neurons with ChR2 co-cultured with myocytes can be optically stimulated to

drive a monolayer of myocytes (Figure S 15 F (i)) and this effect can be blocked using a beta-blocker (metoprolol, Sigma Aldrich, Figure S 15 F (ii)).

(ii) Cardiomyocyte response to ChR2 stimulation with ChR2-expressing neurons as a function of neuron density: Stimulating the ChR2-CSSN (cardiac sympathetic stellate neurons) elicits a rate response in the cardiac monolayer. The number of neurons innervating the myocytes affects the firing frequency of myocytes. Different neuronal plating densities influence the cardiac response. We observe a dose-dependent effect (i.e. the greater the number of neurons innervating the myocytes, the greater the effect; Figure 5 B (ii)).

Firing frequency versus concentration of neurons in co-cultures: There is a fairly strong dose-dependent effect observed, (Figure 5 B (ii)). We observe that most values are >0, indicating a speed up. Where x axis represents ratios of neurons to myocytes (0= myocytes/controls, 1e-05=1:100,000, 0.01=1:100, 0.05=1:20 and 0.2=1:5). No significant increase/decrease observed with light stimulation in controls (no neurons) versus co-cultures. All data analysed using custom-written Matlab script.

(iii) Validation of a co-culture model and translational value- iPSc-CM and iPSc-peripheral neurons:
The data we present are more anecdotal due to the limited n numbers. Example traces, and response to caged nicotine and optogenetic manipulation of iPSc-derived peripheral neurons with immunostains are presented to demonstrate the utility of the methodology presented in this paper. Cardiomyocyte response to ChR2 stimulation with ChR2-expressing peripheral neurons (MOI 2000) as a function of neuron density and blue light protocol of 3 sec on/off protocols were employed. Our data demonstrate the utility and power of our co-culture model and the applicability of high throughput fluorescent techniques (Klimas *et al.*, 2016) for contactless optogenetics experiments required in cell specific perturbations (Figure S 18). Cor.4U® human iPS-derived cardiomyocytes and Peri.4U® human iPS-derived peripheral neurons were grown as described by Axiogenesis handling guides (https://ncardia.com/resources/#manuals).

Here we performed direct adenoviral gene delivery in human iPSc-derived peripheral neurons (ChR2-hiPSc-PN, obtained from Axiogenesis) and observe the cardiac response to light stimulation of neurons (Figure S 18 D), confirming that optogenetic stimulation of co-cultures is a suitable method and alternative for dissecting neural mediated cardiac function in a ‘petri-dish’. To confirm the myocyte-like properties of the iPS-dervied cardiomyocytes, we utilised mouse anti-α-actinin primary antibody and imaged them using a confocal microscope, using the Olympus FluoView FV1000 system. All samples were fixed in 3.7% formaldehyde after performing functional experiments. Cells were permeabilised using 0.02% TritonX-100 for 5 minutes. Antibodies were diluted using 1% bovine serum albumin (Amersham PLC, Amersham, UK). 1% FBS was used as a blocking agent. After antibody staining, cell nuclei were stained with 1 μg/mL DAPI with 10 min incubation in PBS. From gene chip data provided by Ncardia, the iPSc-peripheral neurons Peri.4U have a high expression of Th gene (7719 where 501-1000 = high expression levels); and very low expression levels of ChAT gene (123, where <150 = absent/very low expression). The commercial cardiomyocytes used in the study (Cor.4U [Axiogenesis AG] was believed to be derived from human induced pluripotent stem cells during these studies. After studies had been completed short tandem repeat (STR) testing determined that the cell product was derived from the human embryonic stem cell line RUES2. Regarding Peri.4U; there is no evidence of the use of human embryonic stem cell lines.

5] Measurement of conduction velocity in well-connected SBU cultures with optical mapping: Cardiac myocytes were cultured using the methods described in (Ambrosi *et al.*, 2015) and grown in glass bottom 35mm poly-lysine-coated Petri dishes (bio coat poly-D-lysine, 35 mm Petri plates, Corning; n=6). Neurons were isolated using the same methods described above. Co-cultures were created using different myocyte-neuron concentrations (20:1 n = 6, 100:1 n = 4, 600K:1 n=6). Using conventional optical mapping methods, we measured conduction velocity in well-connected cardiac cultures and co-cultures utilizing the dye Rhod-4-AM (10 µM: AAT Bioquest, Sunnyvale, CA). Rhod-4 AM diluted in Tyrode's solution containing the following in mM: NaCl, 135; $MgCl_2$ 1; KCl, 5.4; $CaCl_2$, 1.5; NaH_2PO_4, 0.33; glucose, 5; and HEPES 5 adjusted to pH 7.4 with NaOH. All experiments were conducted at room temperature. Details for the macroscopic imaging system used in this experiment has been described previously (Jia *et al.*, 2011). Data was spatially and temporally filtered, using the Bartlett and Savistsky-Golay filters, before being analyzed in custom-developed Matlab (Mathworks, Natick, MA) software.

6] Structural studies (confocal microscopy and scanning electron microscopy):
Scanning electron microscopy (SEM): Co-cultures were prepared for SEM using a protocol adapted from (Bozzola, 2007). Briefly, cells were grown on 13 mm glass coverslips and fixed in 2.5% glutaraldehyde, post-fixed in 1% osmium tetroxide at 4°C for 1 hr, taken through an ethanol dehydration series and then dried for 3 minutes with HMDS. Cover-slips were mounted onto SEM stubs with a conductive carbon backing, coated with approximately 8 nm gold and imaged on a JEOL-6390 SEM (Figure 1).

Confocal microscopy: Briefly, cells were grown on 12 mm Poly-D-Lysine/Laminin coated coverslips (BD biosciences, UK) and fixed in 4% paraformaldehyde (Pierce Cat#28906) Following fixation, the cultures were washed in PBS and blocked with 10% goat serum and 0.3% BSA, and permeabilised with 0.1% Triton X-100 for 1 hour at room temperature. Cells were labelled with anti-sacromeric alpha actinin (1:650 A7811, SigmaAldrich, UK), anti-tyrosine hydroxylase (1:250 T1299, Sigma Aldrich), anti-beta2 adrenergic receptor (1:100, ab182136, Abcam, UK) and anti-Vimentin (1:250 ab92547, Abcam, UK) primary antibodies overnight at 4ºC to confirm the presence of sympathetic neurons in co-cultures and to assess the growth of fibroblast in culture with Vimentin. Secondary fluorescent antibodies (Alexa 488 and 647, 1:1000) were then applied for 2 h at room temperature (controls were labelled with secondary antibody only to ensure specificity of labelling, Supplementary Figure S 2 and S 3). Slides were mounted in Vectashield to reduce photobleaching of fluorescence. Cultures were imaged using confocal microscopy (NT confocal laser-scanning microscope, Leica Microsystems Germany).

Measurement of neurite length and calculation of distance between cell boundaries:

SEM image #	neurite length (µm)	number of cell bodies
1	1523.22	1
3	9197.809	5
11	4893.733	5
17	8144.326	8
21	7000.653	7
total	30759.74	26
	average length (µm)	std
	1183.07	375.42

Measuring neurite length: SEM images were calibrated in ImageJ (Schneider et al., 2012) using their scale bars. Using the segmented line tool, neurite lengths were segmented, tabulated, and measured (Supplementary Figure S 1). Cell bodies were classified by: (i) cell body morphology (ii) size and (iii) arborisations confirmed by a Specialist in Anatomy, Prof Helen Christian (Oxford). The average neurite length is computed as a weighted average, 1183.07 ± 375.42 µm, from the data shown in the table above.

Calculating distance between cardiomyocyte cell boundaries: A confocal image stack of fluorescently labelled cardiomyocytes (TH488) was used as the starting point for the image analysis (Fig 2 A (i)). A maximum intensity projection was taken of the image stack before manually segmenting the CM cell boundaries (Fig 2 A(ii)). The image was thresholded and binarised to leave only the cell boundaries on a black background. A computer script then identified random start and end points for transects across this segmented image with a fixed length of 50 microns. All transects starting or terminating outside of the image boundaries were rejected. The intensity profile along each random transect was then used to identify the number of times a cell boundary was crossed ('N'). By dividing 50 µm by N it is possible to estimate the typical distance between cell boundaries for a random transect across the cardiac monolayer.

7] Proteomics:

(i) In-gel trypsin digestion

Cell lysates were mixed with an equal volume of reducing SDS-PAGE sample buffer, heat denatured (95°C for 10 min) and run into the upper part of a Tris-Glycine 8-16% gel (185V, 10m min). Protein material was excised after Coomassie blue staining and cut into 1 – 2 mm^3 gel pieces, which were placed into 1.5 mL sample tubes. Gel pieces were rinsed twice with wash solution for 18h in total (200 µl, 50% methanol, 5% acetic acid). The solutions were removed and gel pieces were dehydrated in acetonitrile (200 µl, 5 min). Supernatant were removed and gel pieces were dried in a vacuum centrifuge for 3 min. Disulfide reduction was performed with 10 mM DTT (30 µl) for 0.5 h, followed by alkylation with 100 mM iodoacetamide (30 µl) for 0.5 h. Supernatants were removed from the gel samples and dehydration with acetonitrile and evaporation performed as described above. Gel pieces were washed with 100 mM ammonium bicarbonate (200 µl, 10 min). Supernatants were removed and dehydration performed with acetonitrile and evaporation as above. The gel samples were then rehydrated on ice with freshly prepared trypsin solution (30 µl, 20 ng/µl sequencing grade trypsin [Promega] in 50 mM ammonium bicarbonate). After rehydration excess trypsin

solution was removed and 50 mM ammonium bicarbonate (10 µl) was added to prevent dehydration of gel pieces. Gel samples were digested at 37ºC for 18h. The gel pieces were then extracted sequentially with 50mM ammonium bicarbonate (60 µl), 50% acetonitrile, 5% formic acid (60 µl) and 85% acetonitrile, 5% formic acid (60 µl). The combined extracts were evaporated in a vacuum centrifuge and were redissolved in 5% acetonitrile, 0.1% formic acid (20 µl) on an ultrasonic bath and transferred into LC-MS sample vials.

(ii) Proteomics analysis by liquid chromatography-tandem mass spectrometry (LC-MS/MS)
Trypsin digested samples were analyzed either on an Amazon Ion Trap mass spectrometer (Bruker Daltonics) as described previously (Kar *et al.*, 2014), or on Q-Exactive (Thermo Scientific) Hybrid Quadrupole-Orbitrap mass spectrometer LC-MS/MS system as detailed below.

Liquid chromatography: A Dionex UltiMate3000 RSLCnano pump at 300 nL/min flow rate was used for analytical separation of digested peptides. A C18, 75 µm x 50 cm (2.6 µm particle size, 150 A; part number: 16126-507569 Thermo Scientific) analytical column was used for separation of peptides and the column temperature was 50°C. Mobile phase used was: A – 0.1% v/v formic acid in water (LC-MS grade) and B – 0.1% v/v formic acid in acetonitrile (LC-MS grade). Two hours linear gradient was used as below, a multistep gradients between 121-132 minutes were used to remove any carryover.

Time	%B	Flow (nL/min)
0-3.1	3	300
3.1-120	40	300
121-125	90	300
126-128	3	300
129-132	90	300
133-158	3	300

A Dionex UltiMate 300 RS pump was used for desalting the peptide. A C18 PepMap (µ-Precolumn, 300 µM I.D. x 5 mm, 100 µm particle size, 100 A; Part number: 160454, Thermo Scientific) trapping column was used for trapping peptides. Buffer used for trapping and desalting peptides was 0.05% v/v Trifluoroacetic acid (TFA) in water (LC-MS grade). 1 µl of samples was injected and allowed for desalting for 3 minutes at 10 µl/min flow rate. After 3 minutes of desalting the trapping valve was changed and directed (backward flush) to analytical column for separation of all trapped peptides on trapping column.

(iii) Mass spectrometry: A bench top Q-Exactive (Thermo Scientific) Hybrid Quadrupole-Orbitrap mass spectrometer was used for data acquisition. A Top10 data dependent acquisition (DDA) method was used. Mass spectrometer was calibrated, for mass accuracy using positive ion calibration mixture provided by mass spectrometer manufacturer, prior to data acquisition. The conditions for the DDA mode were; Chromatographic peak width: 10 s, the Full MS conditions used- resolution: 70,000, AGC target: 1e6, maximum IT (injection time): 100 ms, scan range: 300 to 2000 m/z. The dd-MS2 conditions - resolution: 17,500. The AGC target conditions- 5e4, maximum IT: 100 ms, loop count: 10 (i.e. Top 10), isolation width: 1.6 m/z, fixed first mass: 120.0 m/z, and the data dependent (dd) settings -under fill ratio: 10% (it sets up a minimum intensity threshold of 5e4 ions), charge exclusion: unassigned, 1, 8, >8, peptide match; preferred, dynamic exclusion: 30 s. Normalised Collison Energy (NCE) of 27 was used for fragmentation of peptides in a high-energy collision dissociation (HCD) cell. This method allows the selection, fragmentation and detection of ten precursors in a duty cycle time of 1.42 s.

(iv) Data processing and database searching: For the analyses carried out using the Amazon Ion Trap (Bruker Daltonics), raw LC-MS/MS data were processed and Mascot compatible files were created using DataAnalysis 4.0 software (Bruker Daltonics). Database searches were performed using the Mascot algorithm (version 2.5.1) and the UniProt_SwissProt database with taxonomy restriction 'rat' (v2015.02.04, number of entries 547,357, after taxonomy filter: 7,930). The following parameters were applied: 2+, 3+ and 4+ ions, peptide mass tolerance 0.3 Da, 13C = 2, fragment mass tolerance 0.6 Da, number of missed cleavages: two, instrument type: ESI-TRAP, fixed modifications: Carbamidomethylation (Cys), variable modifications: Oxidation (Met).
For the analyses carried out using the Q-Exactive (Thermo Scientific) Hybrid Quadrupole-Orbitrap mass spectrometer, LC-MS/MS data (.raw files) were converted to .mgf files and Database searches were performed using the Mascot algorithm (version 2.5.1) and the UniProt_SwissProt database with taxonomy restriction 'rat' (v2015.11.26, number of entries after taxonomy filter: 7,954). The following parameters were applied: 2+, 3+ and 4+ ions, peptide mass tolerance 10 ppm, 13C = 2, fragment mass tolerance 0.06 Da, number of missed cleavages: two, instrument type: Q-Exactive, fixed modifications: Carbamidomethylation (Cys), variable modifications: Oxidation (Met).
Protein quantification was performed by spectral counting using the SINQ method (Trudgian *et al.*, 2011). Protein ratios (co-cultures/myocytes) were calculated from SINQ intensities for all quantified protein hits. We chose a 3 -fold change as a threshold for regulation (Protein SINQ Quantitation data provided in Supplementary Tables 5 and 6).

(v) Gene ontology enrichment analysis of the clusters in the network: This was performed for the protein networks obtained by linking the mass spectrometry results (Table 1 and 2) with STRING database.
The following tool was used for the analysis:
https://www.psb.ugent.be/cbd/papers/BiNGO/Home.html
Gene ontology used was from: http://geneontology.org/docs/download-ontology/
The gene ontology has 3 sub-ontologies:

1. Biological Process (BP)
2. Cellular Component (CC)
3. Molecular Function (MF)

We ran over-representation tests for all proteins for all three categories (Supplementary Table 3 and 4).

Supplementary Table 3 has the following structure:

-tab ST3A) 22SDallclusters-BP - this contains the Bingo hypergeometric tests against the Biological Process (BP) gene ontology for 22SD experiment
-tab ST3B) 22SDallcluster-CC - this contains the Bingo hypergeometric tests against the cellular component (CC) gene ontology for 22SD experiment
-tab ST3C) 22SDallcluster-MF - this contains the Bingo hypergeometric tests against the molecular function (CC) gene ontology for 22SD experiment

Supplementary Table 4 has the following structure:

-tab ST4A) SDCN5allclusters-BP - this contains the Bingo hypergeometric tests against the Biological Process (BP) gene ontology for SDCN5 experiment

-tab ST4B) SDCN5allclusters-CC - this contains the Bingo hypergeometric tests against the cellular component (CC) gene ontology for SDCN5 experiment
-tab ST4C) SDCN5allclusters-MF - this contains the Bingo hypergeometric tests against the molecular function (MF) gene ontology for SDCN5 experiment

Insights from Proteomics: Proteomics highlights changes in cytoskeletal, metabolic and nuclear proteins which occur in co-cultures: To further shed light on the molecular mechanisms governing the difference in macroscopic behaviour, we looked at the protein expression profiles between the two cultures. We performed a comparative proteome analysis of harvested cell cultures. Database searches of merged peak list files were carried out using the MASCOT algorithm with taxonomy restriction to rat sequences. False-discovery rate (FDR) estimation was performed through a target-decoy search strategy and all results were displayed at a FDR of ≤1% (Supplementary Table 1).
The identified proteins included many abundant cytoskeletal proteins (actin, tubulin, vimentin, vinculin, actinin), metabolic enzymes (alpha-enolase, GAPDH, pyruvate kinase, ATP synthase) and nuclear proteins (histones H2A, H2B, H3, H4, prelamin) which were detected commonly across the two samples.
Among the most strongly regulated protein hits we see a significant increase in several biological processes (See Figure S 8 and S 10) from 2 independent experiments). From the STRING (Jensen *et al.*, 2009) and Expressence analysis, the most abundant class of proteins in the co-cultures can be seen in Supplementary Table 3 and 4, Enrichment Analysis for two independent samples (SD5 and SD22, significance <0.05). Interestingly, using the Cellular Component analysis (Supplementary Table 3 and 4, tab abbreviated CC), we observe a significant up regulation in translational and mitochondrial related proteins including mitochondrial respiratory chain complex III (Supplementary Table 3 and 4, tab CC, and visualised in Figure S 8 and S 10).

Western blot studies: Western blots of Connexin43 expression in 2 separate culture experiments grown in the absence and presence of sympathetic cardiac stellate neurons (Sprague Dawley (SD) and Wistar Kyoto (WKY) animals), Supplementary Figure S 12. Cell lysate was heated to 95°C for 5 min (to denature the protein) and vortexed before 20 µg was loaded into a 4-12% Tris-Glycine gel. The gel was run for 150 minutes at 125 V. After electrophoretic transfer (2 hours, 40 V) to a membrane (nitrocellulose), the membrane was washed with Tris Buffered Saline (TBS: 50 mM Tris-CI, 150 mM NaCl, pH 7.6) and blocked for an hour using a 5% milk solution (in TBST: TBS with 0.1 % Tween20). The primary antibody (Connexin 43, AB1728, Millipore, UK; 1:200) in 5% milk solution was incubated for 60 min at room temperature on a rocker. The membrane was washed three times in TBST at intervals of 10 minutes and incubated with a horse radish peroxidase (HRP)-conjugated secondary antibody (anti-mouse IgG 1:10000, anti-rabbit IgG 1:10000; both Novus Biologicals) in TBST solution for 60 minutes, then subjected to three washes at 10 minute intervals using TBST. Next, the membrane was washed twice for 10 minutes in distilled water for to ensure complete removal of tween which could interfere with HRP reaction. The ECL+ kit (Perkin Elmer Western Lightning ECL Pro, NEL120001EA) was used and a series of exposures using photographic film was collected. This was repeated with a GAPDH antibody, the loading control, (1:2500, Abcam, ab181602). Optical densitometry of the western blots was conducted using ImageJ software. Connexin43 (Cx43) levels in cultures using Western blot technique (Figure S 12) confirmed that Cx43 was higher in the co-cultures (two independent experiments).

8] Statistics: Details provided in each result and online methods. Data are presented as means +/- stdev. In all cases $P < 0.05$ was considered to indicate a statistically significant difference. The chi-square statistic was used to assess differences in pattern formation. The Kolmogorov-Smirnov test was used to assess normal distribution and subsequent comparison was run using unpaired, two-tailed t-test ($P = 0.026$) for the automated measurement of wavefront speed. The t-test was used to assess patch clamp results. ANOVA followed by Tukey-Kramer was used for well-connected mono and co-culture conduction velocity statistical tests.

References

Ambrosi, C. M., Boyle, P. M., Chen, K., Trayanova, N. A. & Entcheva, E. 2015. Optogenetics-enabled assessment of viral gene and cell therapy for restoration of cardiac excitability. *Sci Rep,* 5**,** 17350.

Ambrosi, C. M. & Entcheva, E. 2014. Optogenetic control of cardiomyocytes via viral delivery. *Methods Mol Biol,* 1181**,** 215-28.

Bozzola, J. J. 2007. Conventional specimen preparation techniques for scanning electron microscopy of biological specimens. *Methods Mol Biol,* 369**,** 449-66.

Burton, R. A., Klimas, A., Ambrosi, C. M., Tomek, J., Corbett, A., Entcheva, E. & Bub, G. 2015. Optical control of excitation waves in cardiac tissue. *Nat Photonics,* 9**,** 813-816.

Ellis-Davies, G. C. 2007. Caged compounds: photorelease technology for control of cellular chemistry and physiology. *Nat Methods,* 4**,** 619-28.

Filevich, O., Salierno, M. & Etchenique, R. 2010a. A caged nicotine with nanosecond range kinetics and visible light sensitivity. *J Inorg Biochem,* 104**,** 1248-51.

Filevich, O., Salierno, M. & Etchenique, R. 2010b. A caged nicotine with nanosecond range kinetics and visible light sensitivity. *Journal of Inorganic Biochemistry,* 104**,** 1248-1251.

Furshpan, E. J., MacLeish, P. R., O'Lague, P. H. & Potter, D. D. 1976. Chemical transmission between rat sympathetic neurons and cardiac myocytes developing in microcultures: evidence for cholinergic, adrenergic, and dual-function neurons. *Proc Natl Acad Sci U S A,* 73**,** 4225-9.

He, Y. & Baas, P. W. 2003. Growing and working with peripheral neurons. *Methods Cell Biol,* 71**,** 17-35.

Hwang, S.-m., Kim, T. Y. & Lee, K. J. 2005. Complex-periodic spiral waves in confluent cardiac cell cultures induced by localized inhomogeneities. *Proceedings of the National Academy of Sciences of the United States of America,* 102**,** 10363-10368.

Jensen, L. J., Kuhn, M., Stark, M., Chaffron, S., Creevey, C., Muller, J., Doerks, T., Julien, P., Roth, A., Simonovic, M., Bork, P. & von Mering, C. 2009. STRING 8--a global view on proteins and their functional interactions in 630 organisms. *Nucleic Acids Res,* 37**,** D412-6.

Jia, Z., Valiunas, V., Lu, Z., Bien, H., Liu, H., Wang, H. Z., Rosati, B., Brink, P. R., Cohen, I. S. & Entcheva, E. 2011. Stimulating cardiac muscle by light: cardiac optogenetics by cell delivery. *Circ Arrhythm Electrophysiol,* 4**,** 753-60.

Kar, P., Samanta, K., Kramer, H., Morris, O., Bakowski, D. & Parekh, A. B. 2014. Dynamic assembly of a membrane signaling complex enables selective activation of NFAT by Orai1. *Current biology : CB,* 24**,** 1361-1368.

Klimas, A., Ambrosi, C. M., Yu, J., Williams, J. C., Bien, H. & Entcheva, E. 2016. OptoDyCE as an automated system for high-throughput all-optical dynamic cardiac electrophysiology. *Nat Commun,* 7**,** 11542.

Larsen, H. E., Lefkimmiatis, K. & Paterson, D. J. 2016. Sympathetic neurons are a powerful driver of myocyte function in cardiovascular disease. *Sci Rep,* 6**,** 38898.

Li, D., Lee, C. W., Buckler, K., Parekh, A., Herring, N. & Paterson, D. J. 2012. Abnormal intracellular calcium homeostasis in sympathetic neurons from young prehypertensive rats. *Hypertension,* 59**,** 642-9.

Macgregor, A., Yamasaki, M., Rakovic, S., Sanders, L., Parkesh, R., Churchill, G. C., Galione, A. & Terrar, D. A. 2007. NAADP controls cross-talk between distinct Ca2+ stores in the heart. *J Biol Chem,* 282**,** 15302-11.

Shcherbakova, O. G., Hurt, C. M., Xiang, Y., Dell'Acqua, M. L., Zhang, Q., Tsien, R. W. & Kobilka, B. K. 2007. Organization of β-adrenoceptor signaling compartments by sympathetic innervation of cardiac myocytes. *The Journal of Cell Biology,* 176**,** 521-533.

Trudgian, D. C., Ridlova, G., Fischer, R., Mackeen, M. M., Ternette, N., Acuto, O., Kessler, B. M. & Thomas, B. 2011. Comparative evaluation of label-free SINQ normalized spectral index quantitation in the central proteomics facilities pipeline. *Proteomics*, 11**,** 2790-2797.

SUPPLEMENTARY FIGURES

Figure S 1: Measuring neurite length in wide field Scanning electron microscopy (SEM) images of co-cultures, related to Figure 1. SEM images were calibrated in ImageJ using their scale bars. Using the segmented line tool, neurite lengths were segmented, tabulated, and measured. Cell bodies were classified by morphology and dendritic arborisation.

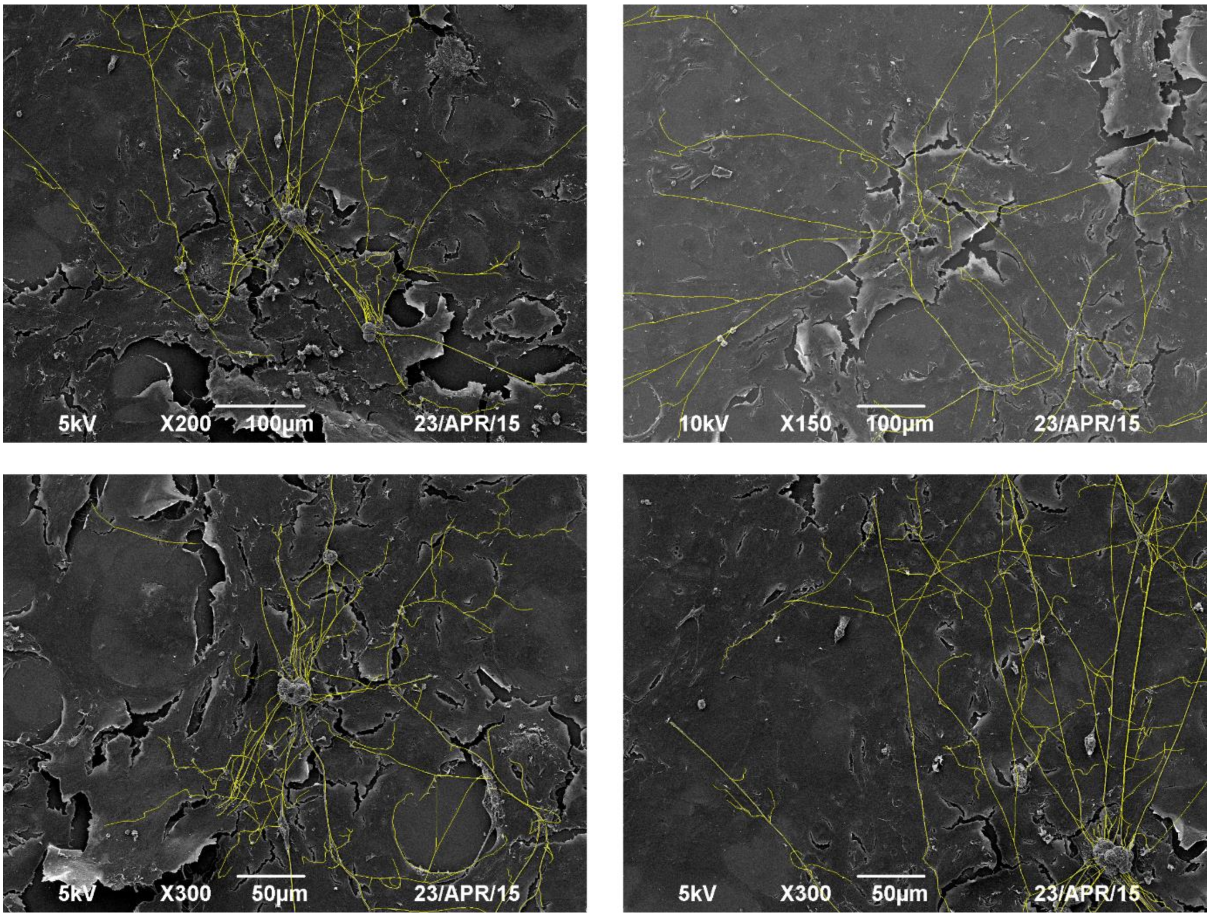

Figure S 2: Co-cultures of ventricular myocytes and sympathetic stellate neurons, related to Figure 1 and 2. Immunofluoresence staining confirmed sympathetic neurons with Tyrosine hydroxylase (TH – red), which is a sympathetic neuron marker. The neuronal processes interweave with the myocytes resulting in a rich innervation of the cardiac myocytes. Th and DAPI (blue) co-staining on co-cultures. Scale bars 20 µm.

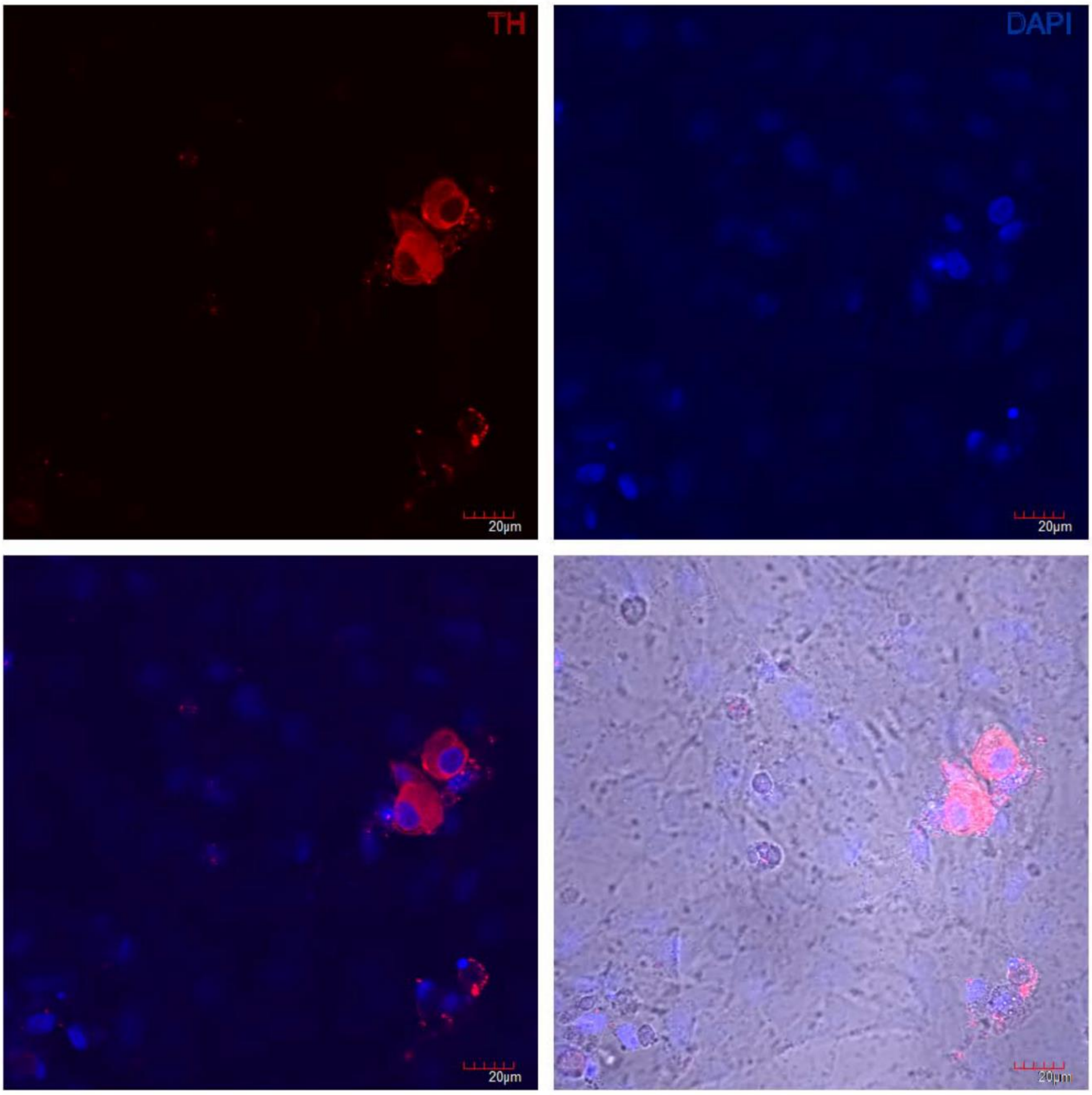

Figure S 3: Co-cultures of ventricular myocytes and sympathetic stellate neurons, related to Figure 1 and 2. Immunofluoresence staining to assess the amount of fibroblasts with Vimentin marker (red), alpha actinin marks the myocytes (green). DAPI (blue) stain on co-culture to identify cell nuclei. Scale bars 20 μm.

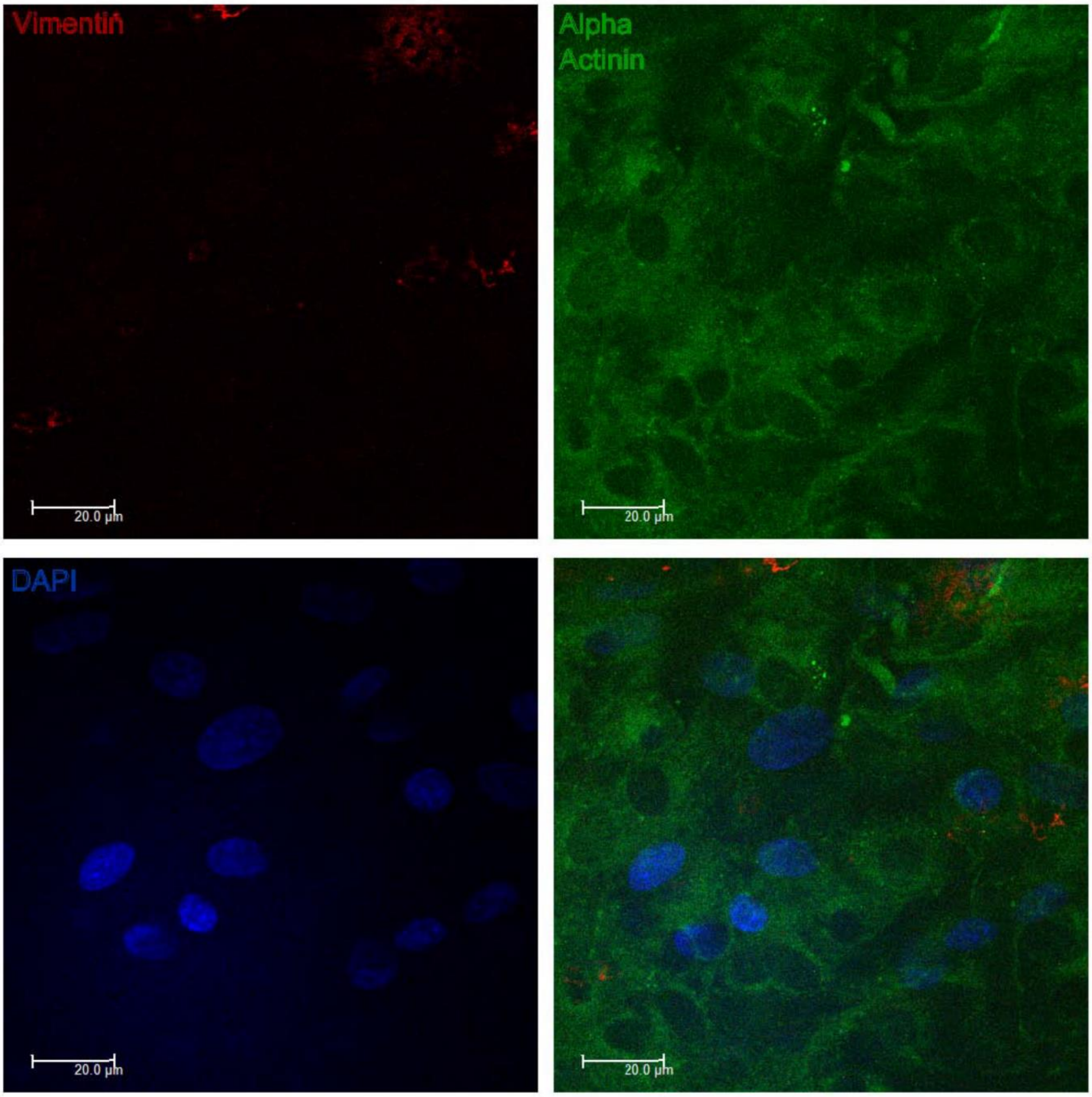

Figure S 4: Co-cultures of ventricular myocytes and sympathetic stellate neurons, related to Figure 1, 2 and 5. Cardiac sympathetic stellate neurons were plated on top of monolayer of ventricular myocytes. Immunofluoresence staining confirmed the sympathetic neurons. Tyrosine hydroxylase (TH – red) is a sympathetic neuron marker. The neuronal processes naturally interweave with the myocytes as seen in the brightfield images resulting in a rich innervation of the myocytes. Immunohistochemistry analyses using TH (red) antibody in the different neuron-myocyte co-culture ratio combinations ((i) 1:5, (ii) 1:20, (iii) 1:100 and (iv) 1:100,000).

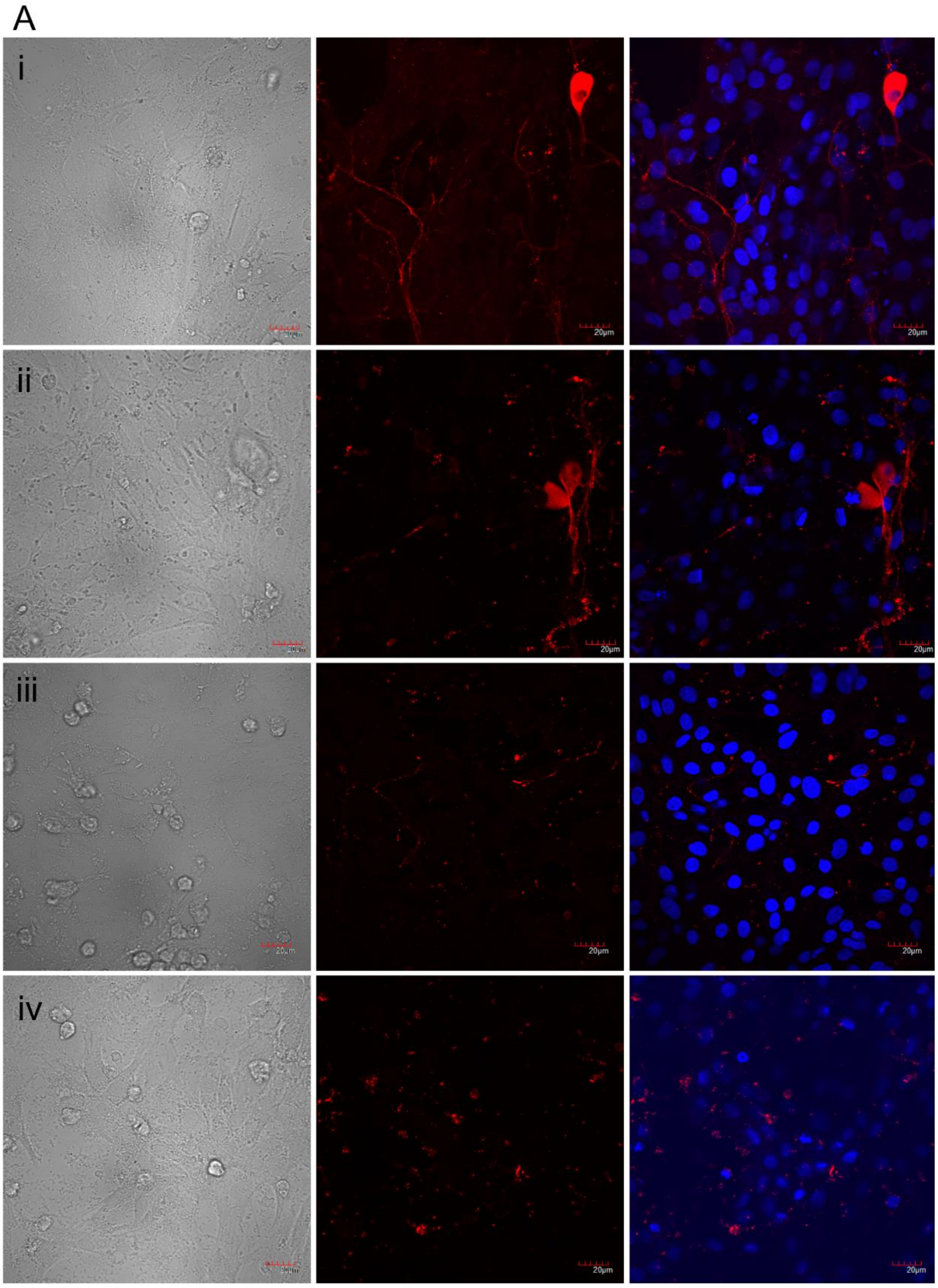

Figure S 5: Off-axis illumination imaging to obtain high contrast images of the monolayer, related to Figure 2 B. Images are processed to calculate the absolute value of the intensity change over n frames. Unfiltered image (Top panel), Note: The image is obtained using a 10x lens instead of the 1x used for the main dye free experiments. (middle and bottom left and right panels) Filter applied to track motion in the cardiac monolayer, traces below, intensity vs time plots for the central 5x5 pixels in the image [Filter: Pt(x,y)Pt-N(x,y)]. The left trace shows intensity from unprocessed images and the right trace show the improved s/n after processing.

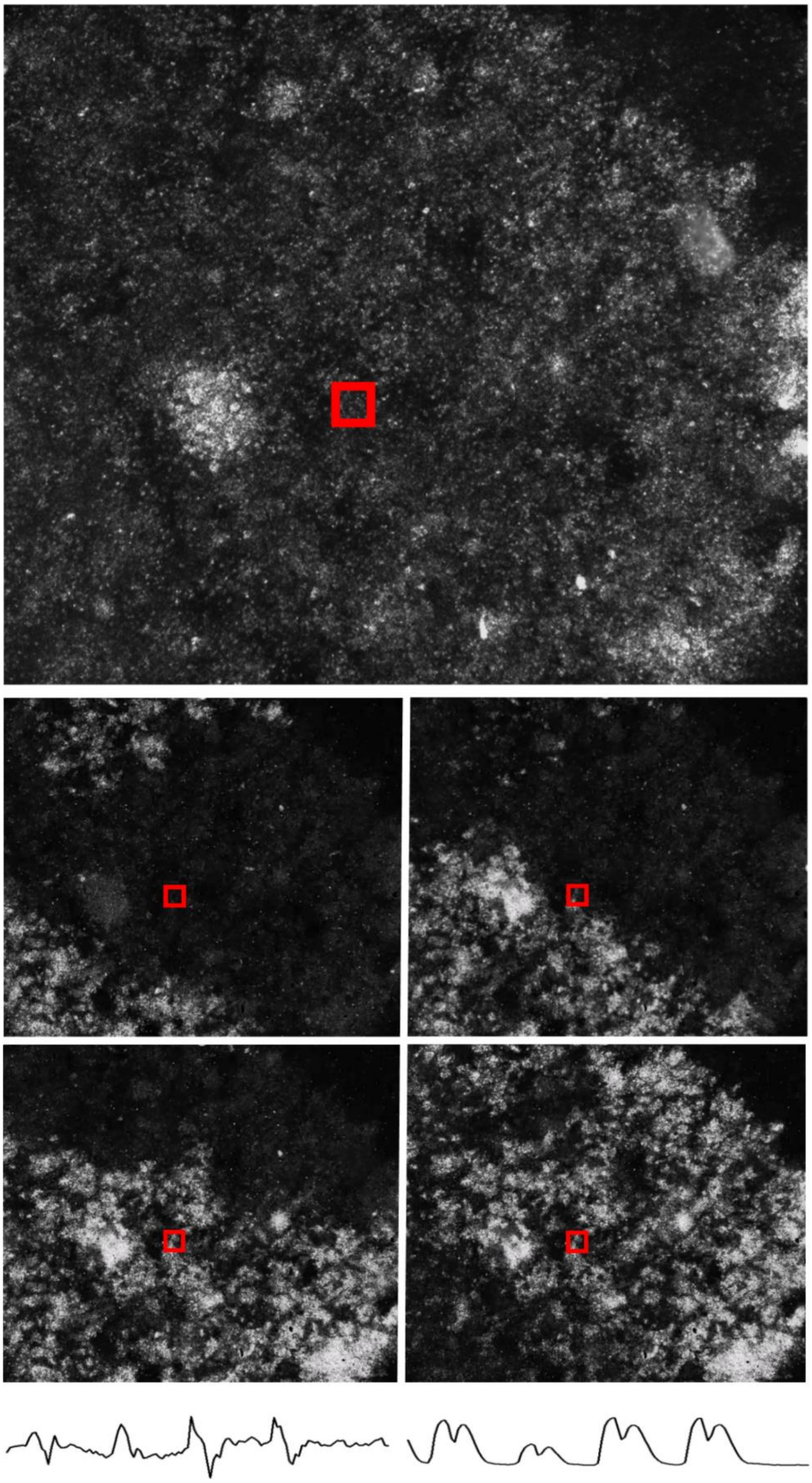

Figure S 6: Individual frames from a video recorded before and after nicotine application, related to Figure 4. The video was processed to show motion transients (white) as described in the supplement. Changes in cardiac macroscopic activity correlate with neuronal bursting following nicotine application. Camera frame rate: 17.5Hz. Each frame pictured represents 119 µm².

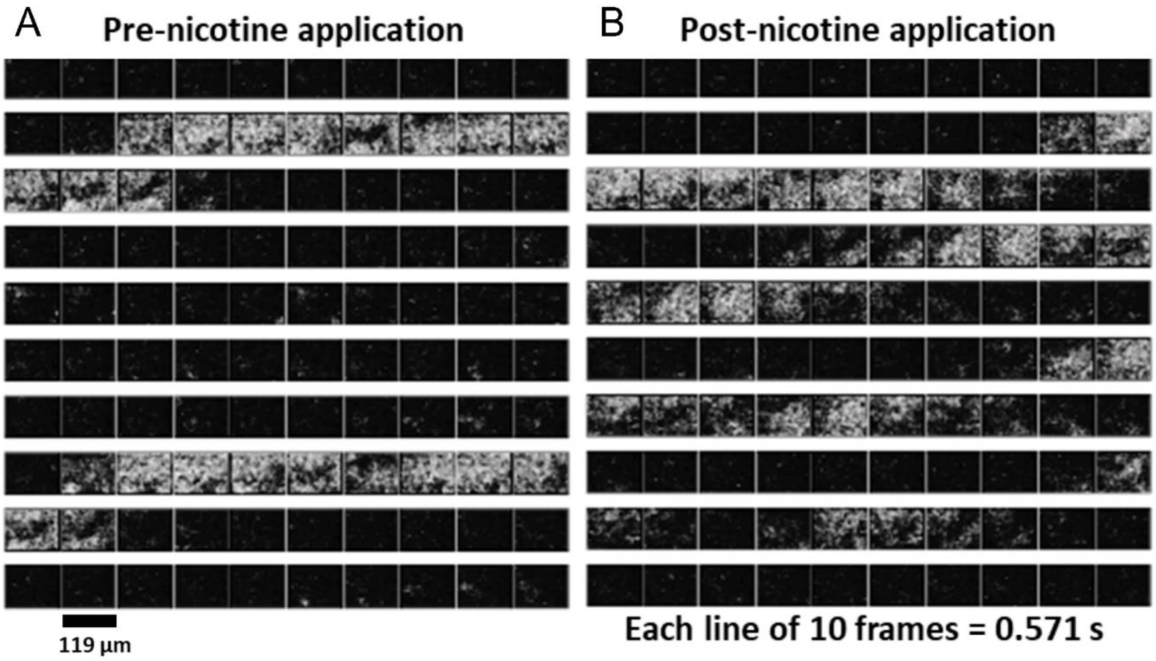

Figure S 7: Quantitative proteomics and Western blot analysis on Oxford co-cultures, related to Figure 2. (A): Representative protein regulation levels in independent experiment SD22, where individual peptides are identified by database search and quantified using spectral counting.

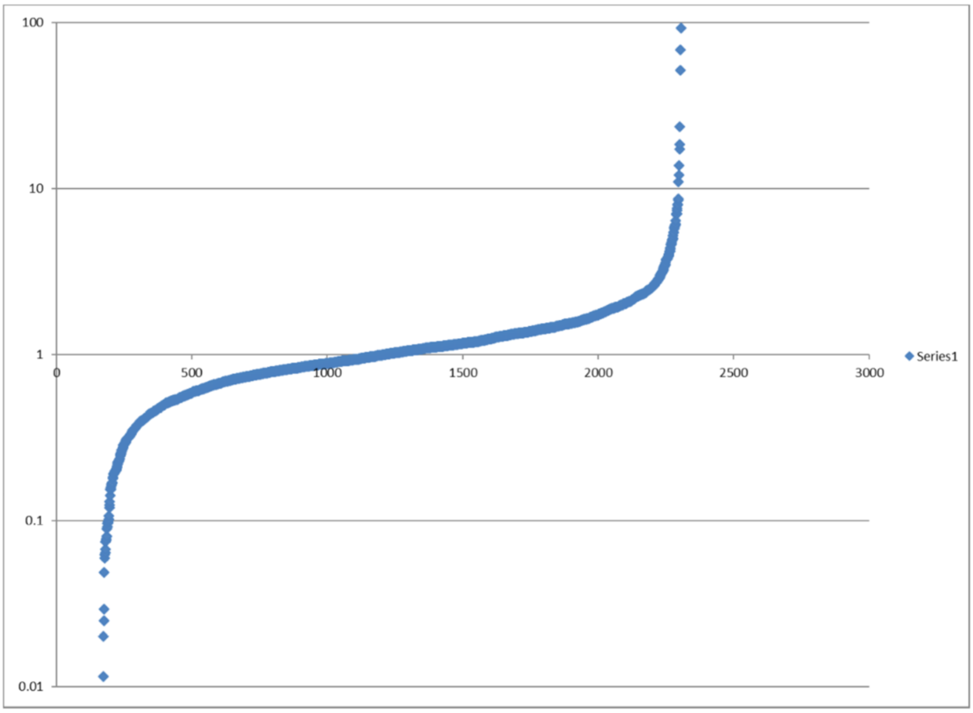

SD22

Figure S 8: Network visualization of Oxford co-cultures from the quantified proteins using Cytoscape (with text mining"/"no evidence" removed), related to Figure 2. Raw protein association data was obtained from STRING. Green nodes represent proteins which are at least 2 fold down-regulated (<0.5), red nodes are proteins which are at least 2 fold up-regulated (>2) and grey nodes are proteins which show less than two-fold variation (between 0.5 to 2). Results for the network condensation analysis using Expressence in SD22 experiment independently. The modules in the figure represent the groups of highest correlated change for the larger network. The node colour is mapped to Measurement values (log transformed MaxQuant intensity values). SD: Sprague Dawley rats.

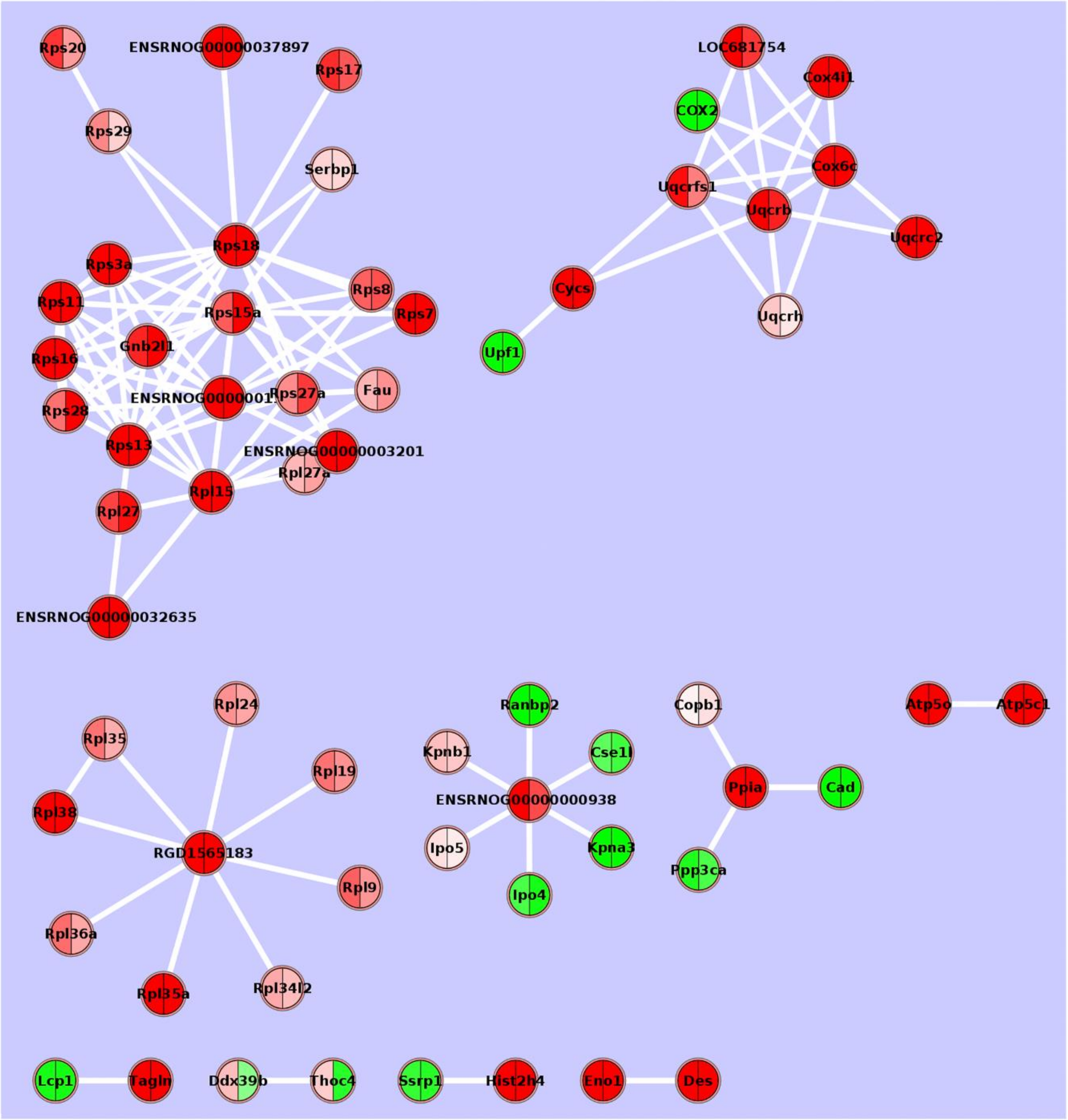

Figure S 9: Quantitative proteomics and Western blot analysis on Oxford co-cultures, related to Figure 2. (A): Representative protein regulation levels in independent experiment SD5, where individual peptides are identified by database search and quantified using spectral counting.

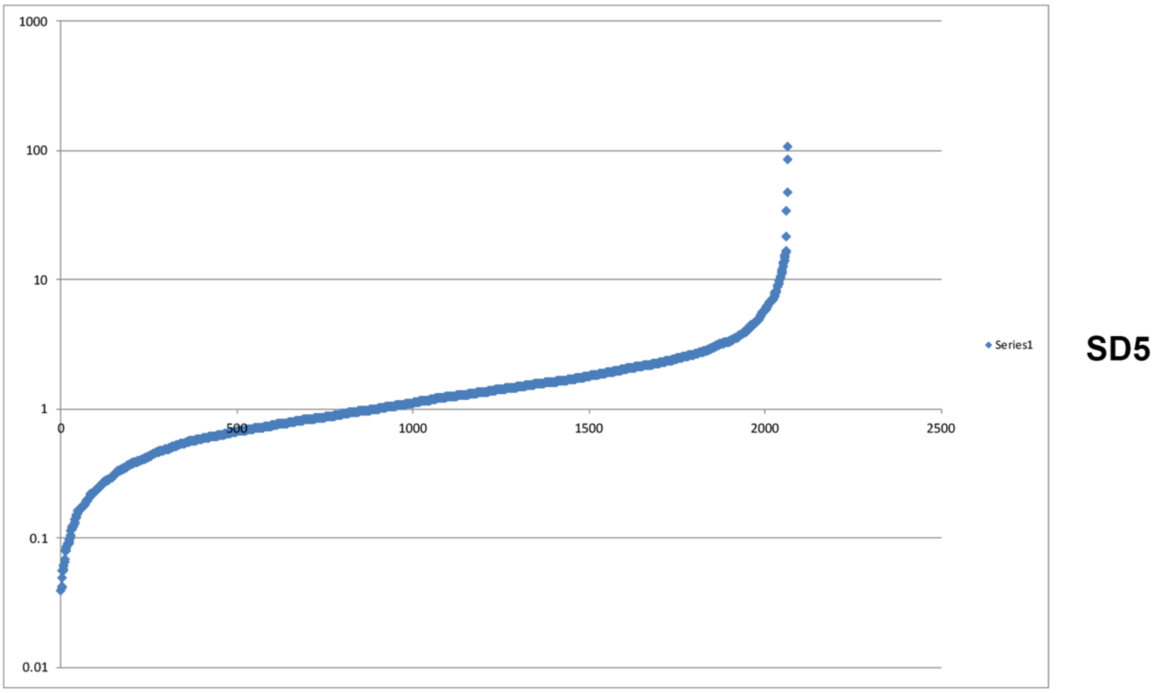

Figure S 10: Network visualization of Oxford co-cultures from the quantified proteins using Cytoscape (with text mining"/"no evidence" removed), related to Figure 2. Raw protein association data was obtained from STRING. Green nodes represent proteins which are at least 2 fold down-regulated (<0.5), red nodes are proteins which are at least 2 fold up-regulated (>2) and grey nodes are proteins which show less than two-fold variation (between 0.5 to 2). Results for the network condensation analysis using Expressence in SD5 experiment independently. The modules in the figure represent the groups of highest correlated change for the larger network. The node colour is mapped to Measurement values (log transformed SINQ intensity values). SD: Sprague Dawley rats.

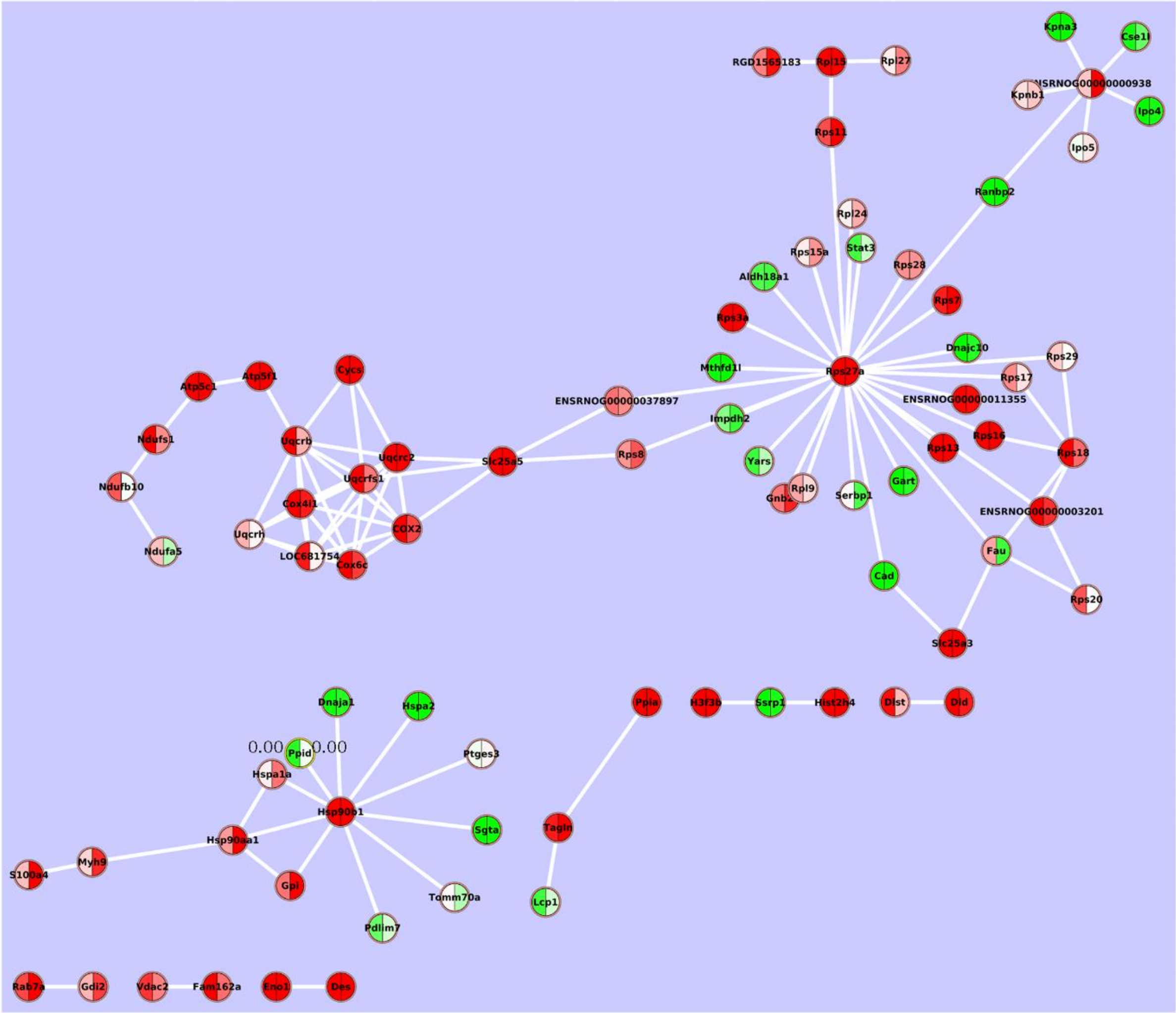

Figure S 11: Western blot analysis and confirmation of protein regulation in two randomly selected proteins from independent Oxford culture experiments (SD22, SD5 and WKY8), related to Figure 2. SD: Sprague Dawley rats, WKY: Wistar Kyoto Rats.

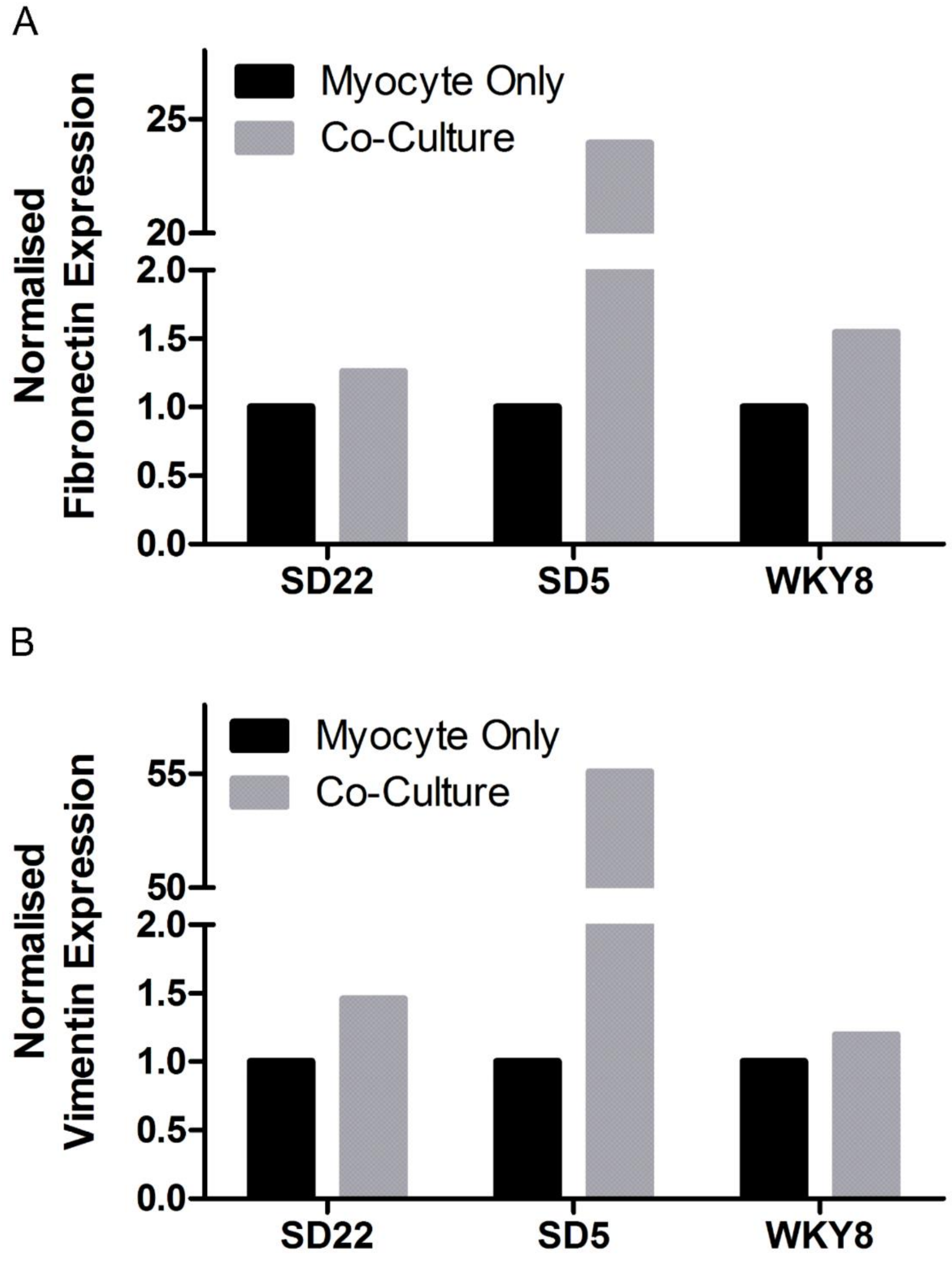

Figure S 12: Western blots of Connexin43 expression in Oxford cultures grown in the absence and presence of sympathetic cardiac stellate neurons in two separate co-culture experiments (SD and WKY), related to Figure 2. SD: Sprague Dawley rats; WKY: Wistar Kyoto Rats.

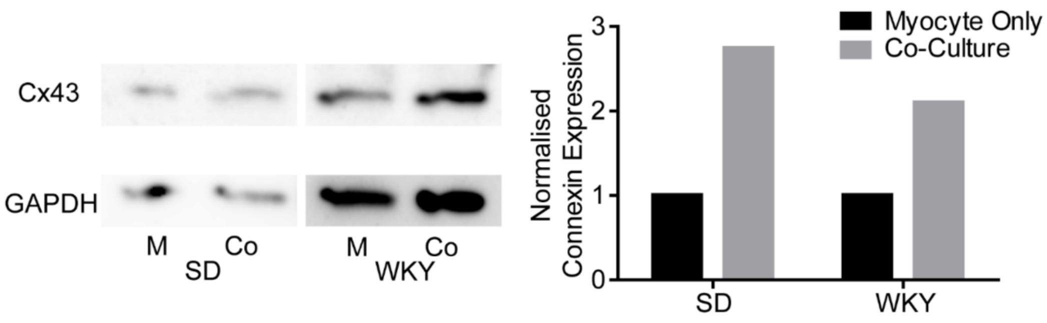

Figure S 13: Effects of sympathetic cardiac stellate neurons on conduction velocity in SBU cardiac/co-cultures using optical mapping (Rhod-4 AM dye) and 1 Hz electrical stimulation, related to Figure 2. Overall, co-culturing myocytes with sympathetic neurons at 3 different concentrations of myocyte-neuron co-cultures did not alter conduction velocity of cardiomyocytes, where average conduction velocities (±std dev) were 17.505 ±2.92 in myocyte only cultures, 17.092 ±4.01 in 20:1 co-cultures, 16.46 ±2.91 in 100:1 co-cultures and 20.791 ±5.79 in 600k:1 co-cultures; (myocyte only cultures n=6, 20:1 co-cultures n=5, 100:1 co-cultures n=4, 600k:1 co-cultures n=6). Plot of conduction velocity of different SBU co-cultures and myocyte only cultures.

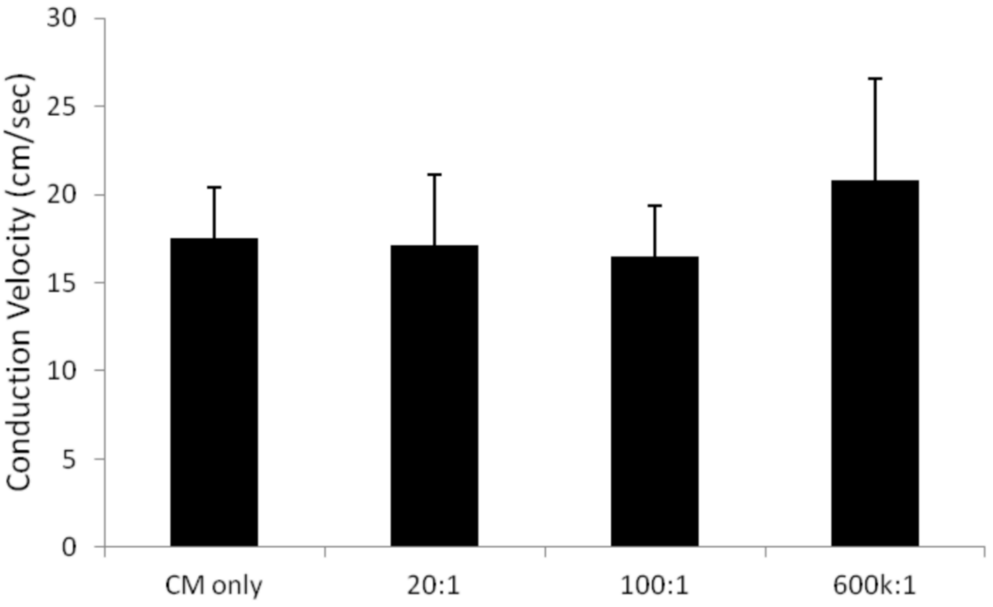

Figure S 14: High throughput fluorescent imaging of co-culture experiments to study the effects of sympathetic cardiac stellate neurons on cardiac activity in well-connected SBU cardiac cultures, related to Figure 5, using optical mapping (cultures loaded with dye Di-4-ANBDQBS). Fishers exact test (two sided), p= 0.0046 statistically significant. n=6 myocytes and n=24 for co-cultures.

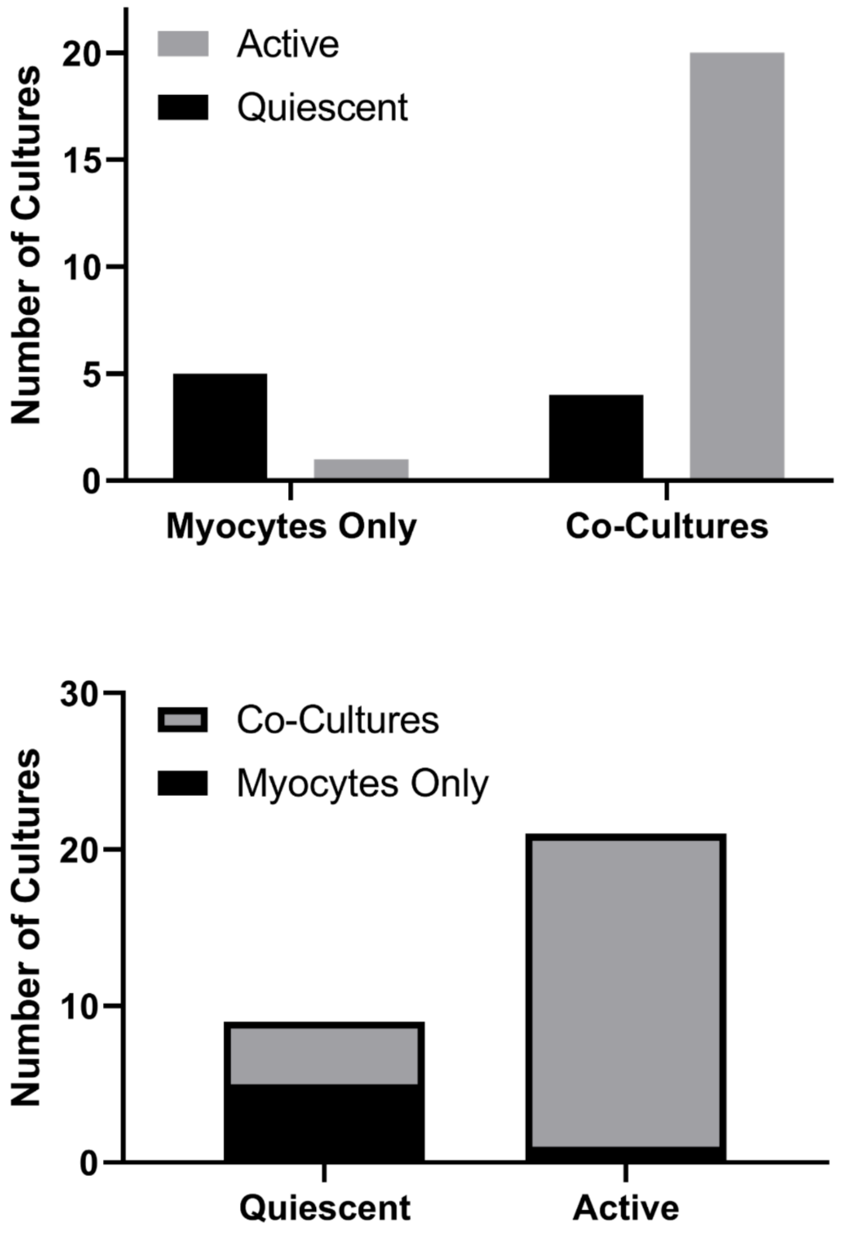

Figure S 15: High throughput fluorescent imaging of SBU co-culture experiments to drive ChR2 stellate sympathetic neurons, related to Figure 5. Co-cultures of neurons and myocytes (loaded with dye Di-4-ANBDQBS spectrally compatible with ChR2). (A and B) Fixed bright field image and YFPChR2 (expressed in the neurons) confocal image of a co-culture (1:5 neuron-myocyte ratio). (C-D) Neuronal stimulation via ChR2, top panel (C) myocyte only culture therefore no beat rate response observed to ChR2 stimulation, bottom panel (D), beat rate response observed during the periods of light stimulation in a co-culture. (E) Post processed traces using custom-written Matlab script. Where, blue is the trace after baseline subtraction and after median filtering, red indicates detected spike times, black is an indicator of when light is present (black down=light off). (F) Neural stimulation via ChR2. (i) Raw traces showing beat rate responses observed in co-cultures, and (ii) no beat rate responses observed in co-cultures that were blocked by the beta blocker metoprolol (10 µM). (iii) Zoomed in region from an action potential response.

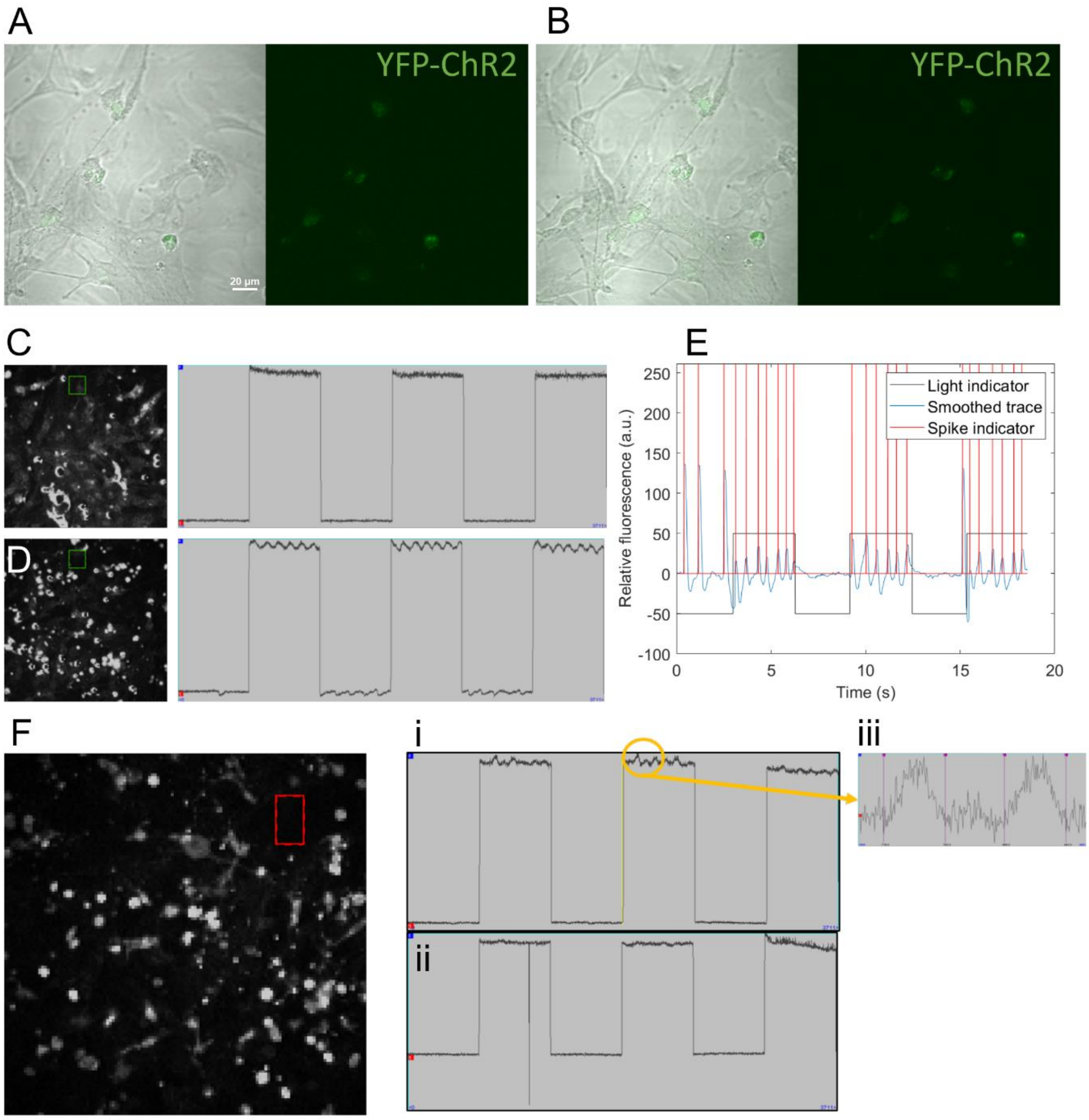

Figure S 16: High throughput fluorescent imaging of SBU cardiac-neuron cultures upon uncaging of nicotine (RuBi-Nicotine from Tocris; related to Figure 5. Panel (A) cardiac only monolayer (control, no neurons). Trace (Ai) full experimental trace, long spike corresponding to flash of light to uncage nicotine. (Aii) Control beat rate prior to uncaging (before light flash); (Aiii) Post-nicotine beat rate after uncaging; (Aiv) Post-nicotine beat rate after 3 minutes. Panel (B) neuron-myocyte co-culture. (Bi) full experimental trace, long spike corresponding to flash of light to uncage nicotine of nicotine; (Bii) Control beat rate prior to uncaging (before light flash); (Biii) Post-nicotine beat rate after uncaging of nicotine; (Biv) Post-nicotine beat rate after 3 minutes. (C-G) Example traces from the different uncaging experiments in the different neuron-myocyte concentration plates. (C) No responses to nicotine were detected in the control myocyte only dishes (representative example). (D) 1:5 neuron-mycoyte co-culture. (E) 1:20 neuron-myocyte co-culture. (F) 1:100 neuron-myocyte co-culture. (G) 1:100,000 neuron-myocyte co-culture. Black line indicates moment of blue light flash to uncage the caged nicotine.

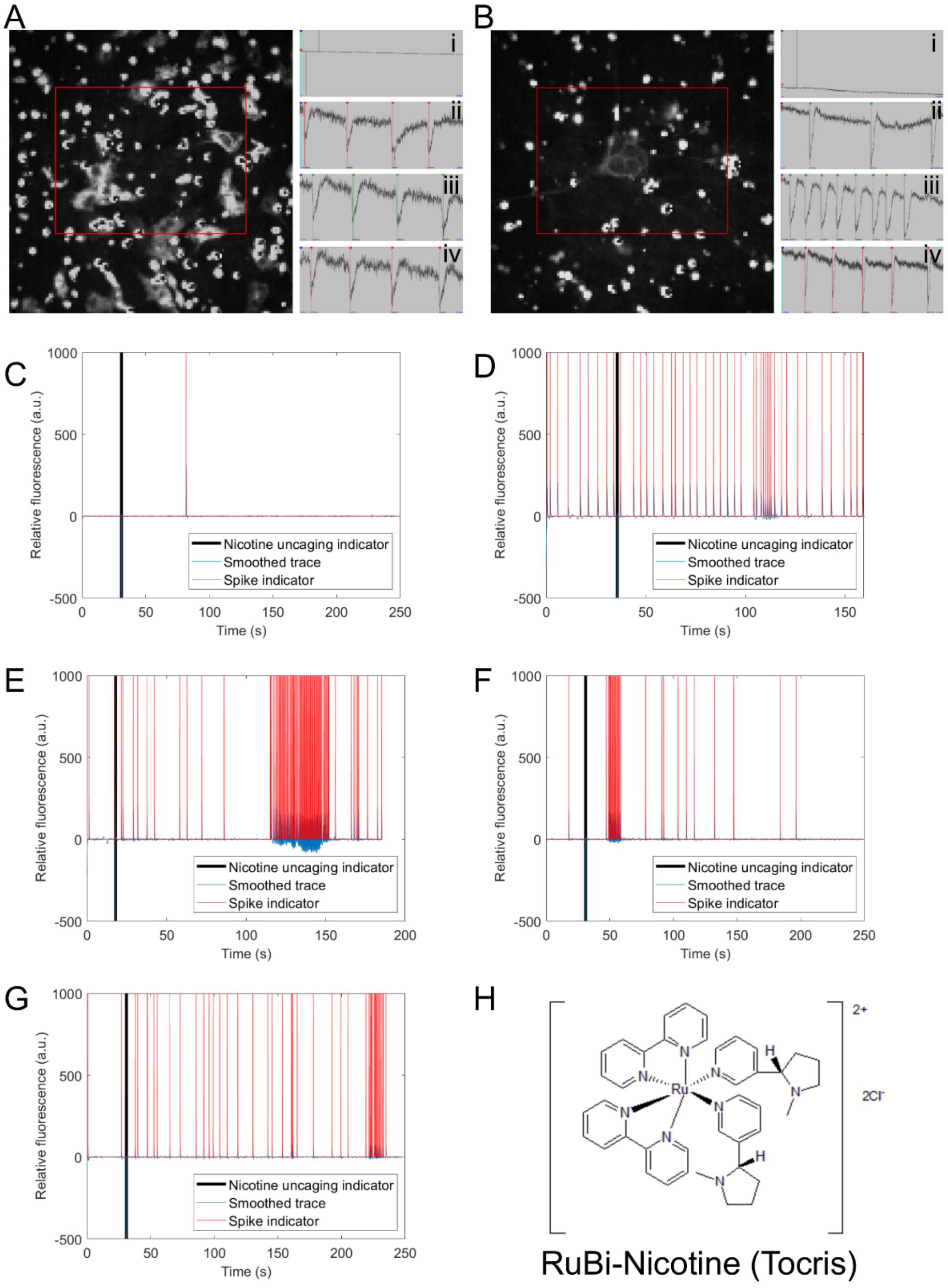

Figure S 17: High throughput fluorescent imaging of SBU co-culture experiments to study the effect of standard nicotine on co-cultures, related to Figure 5. Co-cultures of neurons and myocytes at different neuron to myocyte ratios (A myocyte only, C 1:5, E 1:20, G 1:100 and I 1:100,000) were exposed to 10 µM nicotine (nicotine response traces B, D, F, H and J,) and the voltage traces were recorded (cultures loaded with dye Di-4-ANBDQBS). Example traces and responses are shown in this figure.

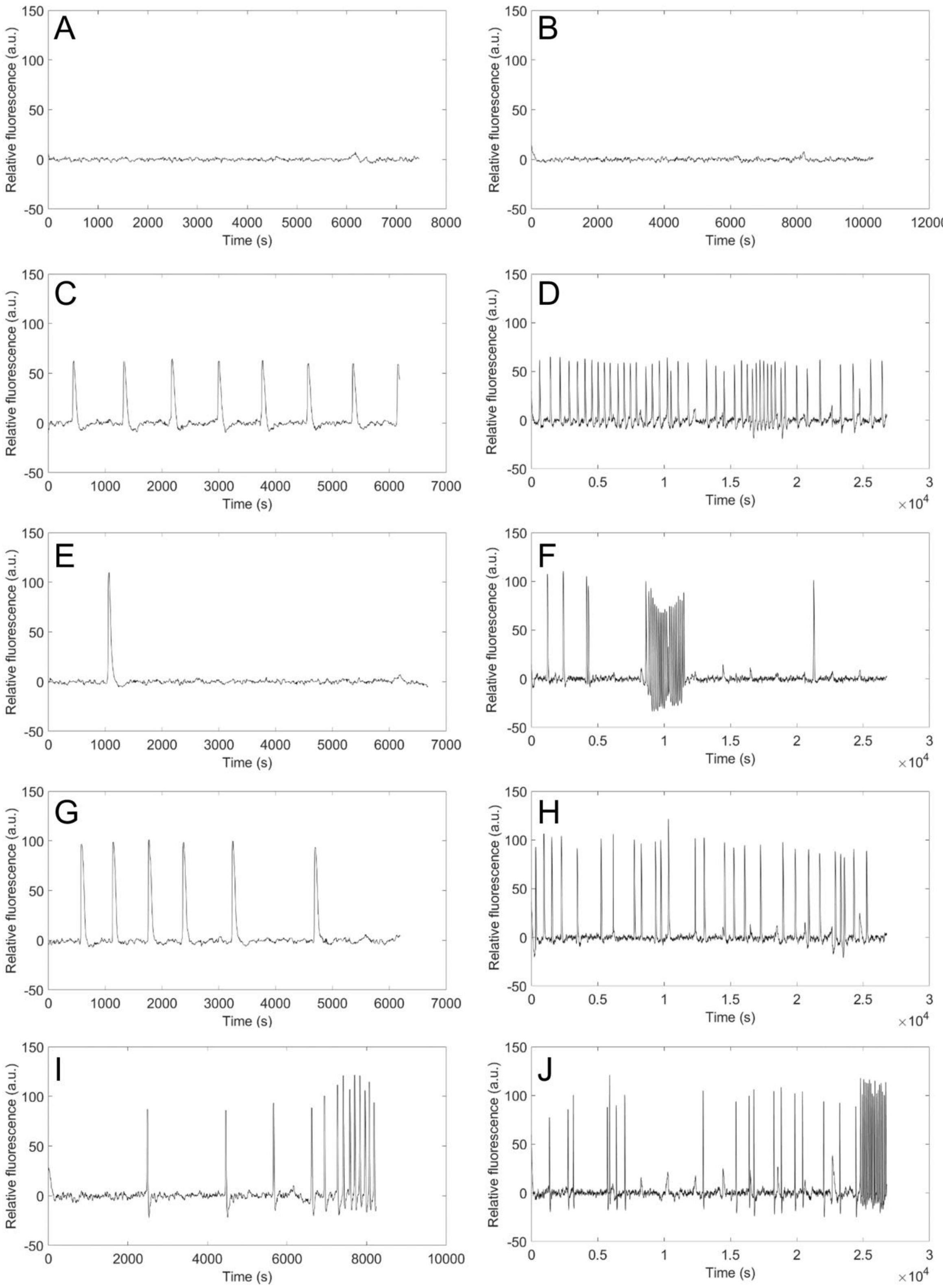

Figure S 18: Pilot co-cultures of Cor.4U cardiomyocytes and Peri.4U hiPSC-derived peripheral neurons (largely sympathetic) developed by Axiogenesis (now Ncardia), related to Figure 5. (A and B) Cardiomyocytes labelled with alpha-actinin staining (green) and DAPI nuclear stain (blue). (C) Peri.4U hiPSC-derived peripheral neurons infected with hChR2-eYFP. (D) Optogenetic neural stimulation of cardiac tissue via Channelrhodopsin2 (ChR2), selectively expressed only in the neurons. Co-cultures of neurons and myocytes (loaded with dye Di-4-ANBDQBS spectrally compatible with ChR2). Optical stimulation (470 nm) was provided at pulse lengths of 3 s, at 0.5 Hz, using irradiance of 0.5-1mW/mm2. Post processed traces using custom-written Matlab software. Traces showing baseline little/no activity and followed by long light pulse stimulation, action potentials are evoked indirectly in the myocytes via the ChR2-light-sensitized neurons. Blue is the trace after baseline subtraction after median filtering, red indicates detected spike times, black is an indicator of when light is present (black up=light off; black down=light on).

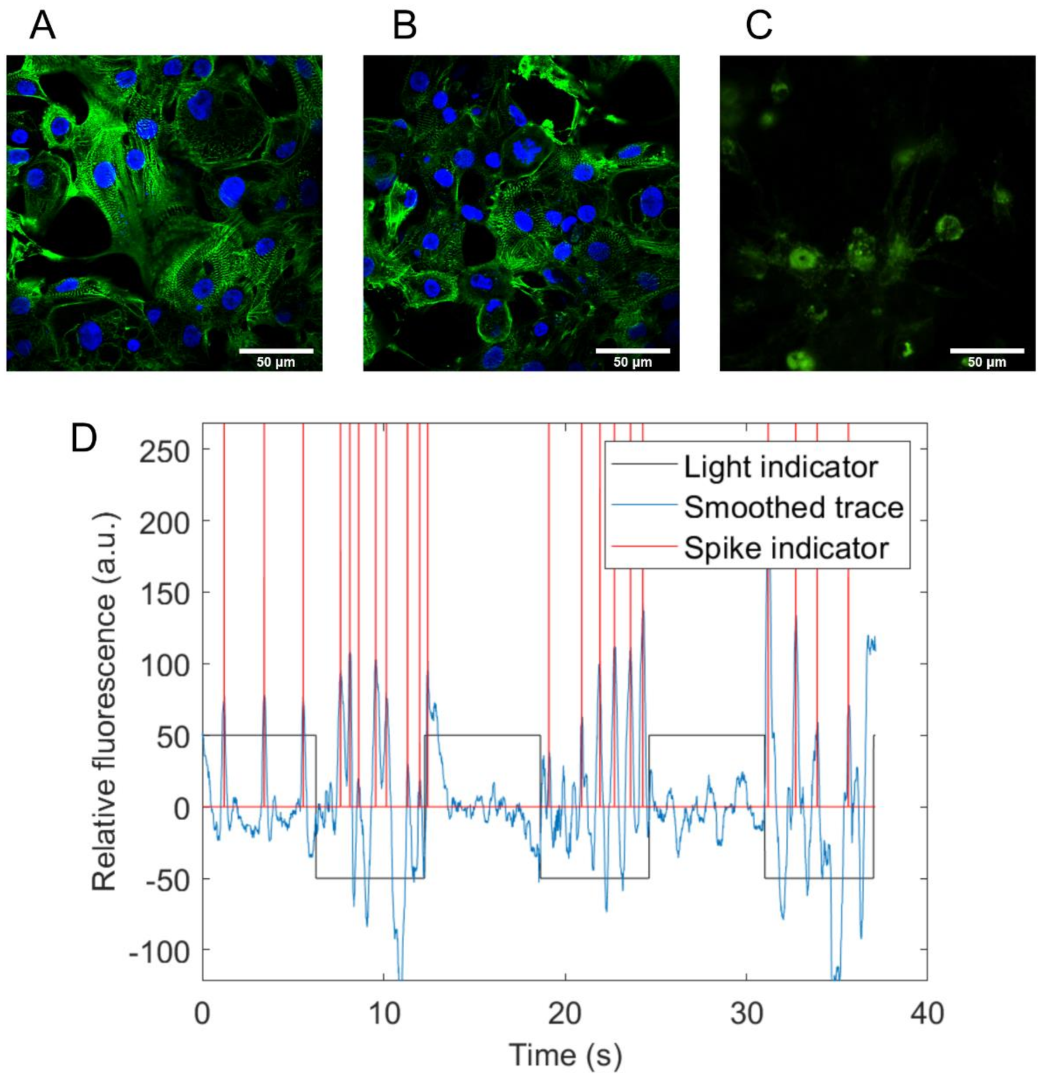

Supplement: Document S1. Transparent Methods and Figures S1–S18 [file mmc1.pdf]
